# *Leonurus japonicus* Houtt. extract containing isoquercitrin reduces airway inflammation in mice with allergic asthma

**DOI:** 10.3389/fphar.2026.1813430

**Published:** 2026-06-18

**Authors:** Camila Carla Guimarães, Marcos De Carvalho Borges, Alexandre Todorovic Fabro, Pedro Henrique Cardoso, Tanize Acunha, Lúcia Helena Faccioli, Sarazete Izidia Vaz Pereira, Silvia Helena Taleb Contini, Suzelei De Castro França, Fabio Carmona, Ana Maria Soares Pereira

**Affiliations:** 1 Faculdade de Tecnologia de Taquaritinga, Centro Estadual de Educação Tecnológica Paula Souza, Taquaritinga, São Paulo, Brazil; 2 Faculdade de Medicina de Ribeirão Preto, Universidade de São Paulo, Ribeirão Preto, São Paulo, Brazil; 3 Departamento de Biodiversidade, Instituto de Biociências, Universidade Estadual Paulista (UNESP), Rio Claro, São Paulo, Brazil; 4 Departamento de Biotecnologia em Plantas Medicinais, Universidade de Ribeirão Preto, Ribeirão Preto, São Paulo, Brazil; 5 Departamento de Análises Clinicas, Toxicológicas e Bromatológicas da Faculdade de Ciências Farmacêuticas de Ribeirão Preto, Universidade de São Paulo, Ribeirão Preto, São Paulo, Brazil

**Keywords:** cytokines, IgE, Lamiaceae, *Leonurus japonicus*, lung function, respiratory disease

## Abstract

**Background:**

*Leonurus japonicus* Houtt. (Lamiaceae) has traditionally been used to treat respiratory disorders, including cough and dyspnea. The aim of this study was to investigate the therapeutic potential of a standardized hydroalcoholic extract from the aerial parts of *L. japonicus* in an *in vivo* model of allergic asthma.

**Methods:**

Balb/c mice were sensitized and challenged with ovalbumin (OVA) and then treated intraperitoneally with *L. japonicus* (at doses of 100, 200, or 400 mg/kg), dexamethasone (at a dose of 2 mg/kg), or saline for seven consecutive days during intranasal OVA challenges. Bronchial hyperresponsiveness, lung cytokine levels (IL-4, IL-5, IL-10, and IFN-γ), inflammatory cells in bronchoalveolar lavage fluid (BAL), lung inflammation, and mucus production were evaluated. The chemical profile of the extract was determined by liquid chromatography coupled with tandem mass spectrometry (LC-MS/MS).

**Results:**

*Leonurus japonicus* significantly reduced bronchial hyperresponsiveness, eosinophil infiltration, peribronchial inflammation and mucus secretion at all tested doses. The 200 mg/kg dose reduced IL-4 and IL-10 levels, and the 400 mg/kg dose decreased IL-5 and IL-10 levels. Isoquercitrin was identified as the major constituent of the extract.

**Conclusion:**

These findings support the traditional use of *L. japonicus* and suggest its potential as an anti-inflammatory and immunomodulatory agent for treating allergic asthma.

## 1 Introduction

*Leonurus japonicus* Houtt. (Lamiaceae) is an annual or biennial herbaceous species native to diverse regions of Asia, including China, Korea, Japan, and Cambodia. However, it has escaped cultivation and become naturalized in other parts of the world, such as South and North America, Europe, and Africa. In Brazil, it is popularly known as “erva-macaé” (Shang et al., 2014; Chen et al., 2024).

A wide range of biological activities has been attributed to *L. japonicus*, including anti-inflammatory (Lee et al., 2020; Wei et al., 2023; Zhang et al., 2023), diuretic (Zhang et al., 2023) and wound healing (de Assunção Morais et al., 2023). These beneficial effects are attributed to the plant’s diverse phytochemical profile. More than 130 chemical constituents have been isolated and identified in *L. japonicus*, including various classes of bioactive compounds such as phenylethanoid glycosides, and sesquiterpene glycosides, alkaloids, flavones and diterpenes (Li et al., 2024).

*Leonurus japonicus* is morphologically related to *Leonurus sibiricus* L., and the two species have often been misidentified and taxonomically confused. In China, for instance, *L. sibiricus* is used similarly to *L. japonicus* in traditional medicine (Huang et al., 2024). The morphological differences between these two taxa were clarified by Harley and Paton (Harley and Paton, 2001) who noted that *L. japonicus* has a smaller calyx with erect teeth. Cytogenetic and biochemical analyses further distinguish them: *L. japonicus* has 2n = 20 chromosomes and accumulates leonurine predominantly in its aerial parts (with trace amounts in the roots), while *L. sibiricus* has 2n = 18 chromosomes and lacks detectable leonurine in any tissue (Yang et al., 2022).

*Leonurus japonicus* is the only species of the genus found in Brazil, where it is widely distributed in anthropized areas (Antar, 2025). In contrast, *L. sibiricus* is native to and largely restricted to Asia (Harley and Paton, 2001). However, misapplication of the name *L. sibiricus* persists in several Brazilian herbaria (Antar, 2025), suggesting that many Brazilian studies referencing *L. sibiricus* may actually refer to *L. japonicus*. Despite this confusion, both species have been traditionally used in folk medicine to treat various health conditions, such as flu, cough, dyspnea, and bronchitis (Parente and Rosa, 2001; Bratti et al., 2013).

*Leonurus japonicus* has shown anti-inflammatory and immunomodulatory potential in respiratory diseases, as demonstrated in both experimental and clinical studies. In a murine model of PM10D-induced airway inflammation, its extract reduced reactive oxygen species (ROS) and inhibited NF-κB pathway activation in alveolar macrophages. When combined with *Lactiplantibacillus plantarum* KC3, it suppressed neutrophil infiltration, decreased COX-2 expression, modulated gut microbiota, and upregulated immunoregulatory genes such as Foxp3, TGF-β1, and IL-10, indicating systemic anti-inflammatory effects (Shin et al., 2024). In an asthma model, trigonelline, isolated from *L. japonicus*, reduced mast cell degranulation and inflammatory mediators (PGD2, LTC4), with HIF-1α identified as a potential target (Zhang et al., 2021). These results highlight *L. japonicus* as a promising source of anti-inflammatory agents, especially for immune-mediated diseases like asthma.

Additionally, in a randomized, placebo-controlled clinical trial involving adults with respiratory discomfort, the combination of *L. japonicus* and *L. plantarum* KC3 was well tolerated, improved health-related quality of life and dyspnea, and reduced inflammatory cytokines, although it did not significantly change pulmonary function (Kim et al., 2024).

Asthma is a heterogeneous disease affecting approximately 300 million people worldwide (GBD 2021 Asthma and Allergic Diseases Collaborators, 2025), with increasing incidence in low- and middle-income countries (Porsbjerg et al., 2023). Although various pharmacological treatments are available and most patients can achieve an adequate asthma control, undertreatment remains common, therapeutic response can be inconsistent for a considerable number of patients, and access to effective care is often limited, especially in underserved regions (Porsbjerg et al., 2023; Caminati et al., 2021). Therefore, satisfactory asthma control still remains a challenge worldwide (Caminati et al., 2021). These challenges have contributed to a growing interest in identifying effective alternative and complementary therapies, including plant-based medicines.

Previous studies by our research group have shown that several species such as *Erythrina mulungu* (Amorim et al., 2018), *Eclipta prostata* (Morel et al., 2017; Morel et al., 2024), *Uncaria tomentosa* (Azevedo et al., 2018), *Pyrostegia venusta* (Balestra et al., 2021), *Momordica charantia* (Frota et al., 2022) and *Stachytarpheta cayennensis* (Guimarães et al., 2023), possess anti-inflammatory and antioxidant properties, and have been shown to decrease bronchial hyperresponsiveness *in vivo*, contributing to asthma control in experimental models. These findings support the therapeutic potential of herbal medicines to enhance treatment outcomes while minimizing adverse effects (Manarin et al., 2019; Shakerinasab et al., 2024).

Considering its traditional use and bioactive composition, this study aimed to investigate, for the first time, the antiasthmatic effects of a hydro-alcoholic extract of *L. japonicus* aerial parts using a murine model of ovalbumin-induced allergic asthma.

## 2 Materials and methods

### 2.1 Ethical aspects

All stages of animal study were conducted at Faculty of Medicine of Ribeirão Preto, University of São Paulo (FMRP-USP) in accordance with the National Council for Animal Experimentation Control (CONCEA) guidelines. The study was approved by the Ethics Committee on Animal Research at (FMRP-USP; protocol no. 181/2018, approved on 25 February 2019). The use of plant material was authorized by the National Council for Scientific and Technological Development (CNPq), on behalf of the Genetic Heritage Management Council/Ministry of the Environment (protocol no. ABC8314).

### 2.2 Plant material

Aerial parts of *L. japonicus* were collected on 13 May 2019, at the Jardim Botânico de Plantas Medicinais Ordem e Progresso (Jardinópolis, SP, Brazil). The plant material was authenticated by Pedro Henrique Cardoso (Escola Nacional de Botânica Tropical, Instituto de Pesquisas Jardim Botânico do Rio de Janeiro, Rio de Janeiro, Brazil). A voucher specimen was deposited in the in the Herbarium of Medicinal Plants at UNAERP (number 1916).

### 2.3 Extract preparation

The plant material was dried in a circulating-air oven at 45 °C for 72 h, then pulverized and passed through a 40-mesh sieve. The hydro-alcoholic extract of *L. japonicus* was prepared at a concentration of 10% (w/v) by macerating 695 g of the powdered material in a mixture of 7:3 ethanol and distilled water (v/v) for 7 days. The extract was then filtered, concentrated by rotary evaporation, and subsequently lyophilized.

### 2.4 Animals and experimental design

Male BALB/c mice (6–8 weeks old and weighing 20–30 g) were obtained from the animal breeding facility at FMRP-USP and housed under standard conditions with *ad libitum* access to food and water.

Animals were sensitized with intraperitoneal (IP) injections of isotonic saline solution (200 µL) containing ovalbumin (OVA, 10 μg, Sigma grade V, Sigma-Aldrich) and aluminum hydroxide (1 mg) on two occasions, 1 week apart. One week after the second sensitization, mice were challenged on four alternate days via nasal instillation of OVA solution (50 µL) under light isoflurane anesthesia (Isoforine™; Cristália Produtos Químicos e Farmacêuticos). Control groups received isotonic saline solution.

Mice were divided into 6 groups (6–8 per group): (i) SAL-SAL, challenged and treated with saline; (ii) OVA-SAL, challenged with OVA and treated with saline; (iii) OVA-LJ100, challenged with OVA and treated with *L. japonicus* extract at 100 mg/kg; (iv) OVA-LJ200, challenged with OVA and treated with *L. japonicus* extract at 200 mg/kg; (v) OVA-LJ400, challenged with OVA and treated with *L. japonicus* extract at 400 mg/kg; (vi) OVA-DEXA, challenged with OVA and treated with dexamethasone (2 mg/kg; Hipolabor). All treatments were administered IP.

### 2.5 Assessment of bronchial hyperresponsiveness

Twenty-four hours after the last OVA challenge bronchial hyperresponsiveness was assessed, as previously described (Borges et al., 2013). The animals were anesthetized (10 mg/kg xylazine and 100 mg/kg ketamine; Syntec), tracheostomized, and connected via a cannula to a flexiVent™ (SCIREQ) small animal ventilator. Ventilation was set to 150 breaths/min with a positive end-expiratory pressure (PEEP) of 3 cm H_2_O. Pancuronium bromide (1.2 mg/kg) was administered IP to induce paralysis. After, respiratory parameters, resistance (Rrs), elastance (Ers), tissue damping (G), and tissue elastance (H), were assessed under basal conditions and after exposure to saline containing increasing concentrations of methacholine (0, 6.25, 12.5, 25, and 50 mg/mL) administered by an ultrasonic nebulizer (Hudson RCI). Only curves with coefficients of determination ≥0.90 were considered.

### 2.6 Collection and analysis of bronchoalveolar lavage fluid (BAL)

Following ventilation, two BAL samples were obtained from each animal by sequentially instilling and aspirating 1 mL of saline through the trachea. The samples were centrifuged, and the resulting pellets were resuspended in 500 μL of isotonic saline. An aliquot was taken to determine the total cell number using trypan blue exclusion and a hemocytometer. The remaining suspension was subjected to Cytospin preparation (Thermo Fisher Scientific) and stained with the rapid Panoptic method (Laborclin) for differential cell analysis. For each animal, 300 inflammatory cells were counted (Morel et al., 2017).

### 2.7 Cytokines and IgE quantification

Blood samples were obtained from the right ventricle by direct puncture. Serum anti-OVA IgE levels were determined following previously described protocols (Prado et al., 2015). After the *in vivo* assessments, mice were euthanized and lung tissues were collected. The right lungs were processed for cytokine determination according to established procedures (Amorim et al., 2018). Concentrations of interleukin 4 (IL-4), IL-5, IL-10, and IFN-γ were measured using BD OptEIA™ ELISA kits (BD Biosciences).

### 2.8 Histological and morphometric analysis

The left lung was inflated with 10% neutral buffered formalin (25 cm H_2_O, 25 min), fixed for 24 h, paraffin-embedded, sectioned (4 μm), and stained with hematoxylin-eosin (H&E) or periodic acid–Schiff (PAS). Sections were examined under a Leica DM500™ microscope (200×; Leica Microsystems). For each animal, five to six bronchioles with intact epithelium and a diameter ratio ≥0.5 were selected for morphometric evaluation of peribronchial inflammation (H&E) and mucus production (PAS). Inflammation and mucus scores were determined as described by Weibel (Weibel, 2010), using a 50-line/100-point grid superimposed on each image, and expressed as the ratio of line intersections with inflammatory or mucus cells to the total number of lines in the peribronchial region (Mühlfeld and Ochs, 2013). Inflammation and mucus scoring were performed by a single unblinded scorer.

### 2.9 Isolation and structural identification of isoquercitrin

The procedures for the isolation and structural identification of isoquercitrin from the hydro-alcoholic extract of *L. japonicus* aerial parts, including chromatographic separation and nuclear magnetic resonance (NMR) spectral analysis, are detailed in the Supplementary Material (Section B — NMR section).

### 2.10 Phytochemical characterization

The Acquity XSelect HSS T3 column was selected for the untargeted LC-QTOF-MS/MS profiling because its trifunctional C18 ligand with polar endcapping enables simultaneous retention of compounds with widely different polarities, from polar phenolic acids and amino acid derivatives to flavonol glycosides and methyl ethers, in a single run, making it well suited for the comprehensive (Level 2, Schymanski et al., 2014) annotation of complex plant extracts. To complement this discovery-mode approach with an orthogonal, targeted confirmation of isoquercitrin, the major bioactive compound, a second analysis was carried out on a conventional reverse-phase Ascentis Express C18 column coupled to UV diode array detection (DAD) and a Xevo TQ-S triple-quadrupole mass spectrometer; this two-tier strategy provides independent retention-time, UV-spectrum and tandem-MS evidence on a chromatographic phase different from the QTOF analysis, in agreement with current reporting standards in natural-product metabolomics. To confirm the presence and retention characteristics of isoquercitrin, the extract and a commercial isoquercitrin standard were analyzed using an ACQUITY UPLC H-Class system (Waters Corporation) for ultra-performance liquid chromatography (UPLC) coupled to a diode array detector (DAD) and Xevo TQ-S tandem quadrupole mass spectrometer (Waters Corporation, Milford, MA). The system operated with a Z-spray ionization source in both positive and negative modes. Samples were solubilized in methanol (1.0 mg/mL); the stock solution diluted to 50 μg/mL, and injected (5 µL) into an Ascentis® Express C18 HPLC column (100 × 4.6 mm, 2.7 µm particle diameter) from Supelco. The mobile phase consisted of 0.1% formic acid (solvent A) and methanol +0.1% formic acid (solvent B). The gradient started at 30% B, increased to 90% B over 15 min, held for 5 min, and returned to 30% B over the next 5 min with time of 26 min and a flow rate of 300 μL/min. Compounds were monitored using a diode array detector (DAD) in the wavelength range of 220 to 700 nm. Data analysis was performed using MassLynx v 4.1.

### 2.11 Statistical analysis

Data were analyzed using GraphPad Prism 5.0 (GraphPad Software, La Jolla, CA, USA). Two-way analysis of variance (ANOVA) followed by Bonferroni post-test was applied to evaluate bronchial hyperresponsiveness to increasing concentrations of methacholine (Mch). One-way ANOVA with Bonferroni post-test was used for comparisons among experimental and control groups for other outcomes. Statistical significance was considered at P < 0.05.

## 3 Results

### 3.1 Effect of *L. japonicus* on bronchial hyperresponsiveness (BHB)

**FIGURE 1:**
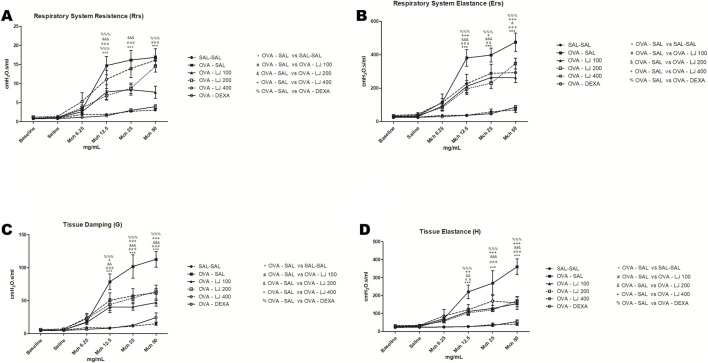
Effects of a hydro-alcoholic extract of *Leonurus japonicus* on the bronchial hyperresponsiveness of ovalbumin (OVA)-challenged mice measured by methacholine (Mch) challenge test (6.25–50 mg/mL). **(A)** Respiratory system resistance (Rrs), **(B)** respiratory system elastance (Ers), **(C)** tissue damping (G), and **(D)** tissue elastance (H). OVA-SAL: sensitized and challenged with OVA and treated with saline solution; SAL-SAL: sensitized, challenged and treated with saline solution; OVA-LJ100, OVA-LJ200, OVA- LJ400: sensitized and challenged with OVA and treated with 100, 200 and 400 mg/kg of LJ, respectively; OVA-DEXA: sensitized and challenged with OVA and treated with 2 mg/kg dexamethasone. Values are shown as mean ± SEM (n = 8 per group). OVA-SAL vs. SAL-SAL - P < 0.001 (***); OVA-SAL vs. OVA-DEXA - P < 0.001 (%%%); OVA-SAL vs. OVA-LJ100 - P < 0.01 (##) and P < 0.001 (###); OVA-SAL vs. OVA-LJ200 - P < 0.05 (&), P < 0.01 (&&) and P < 0.001 (&&&); OVA- SAL vs. OVA-LJ400 - P < 0.05 (+), P < 0.01 (++) and P < 0.001 (+++).

Respiratory parameters are shown in Figure 1. Ovalbumin (OVA)-challenged mice exhibited a significant increase in BHR compared to saline-challenged mice. Treatment with *L. japonicus* extract at all tested doses significantly decreased BHR compared to the OVA-SAL group (Figure 1). Specifically, the OVA-LJ100, OVA-LJ200, and OVA-LJ400 groups had significantly lower values of respiratory system elastance (Ers), tissue damping (G), and tissue elastance (H) (Figures 1B–D). Respiratory system resistance (Rrs) was significantly reduced in the OVA-LJ100 and OVA-LJ200 groups compared to OVA-SAL group (Figure 1A). Administration of dexamethasone (OVA-DEXA group) also significantly decreased BHR compared to OVA-challenged mice.

### 3.2 *L. japonicus* extract decrease inflammatory cell infiltration in bronchoalveolar lavage (BAL) equations

**FIGURE 2:**
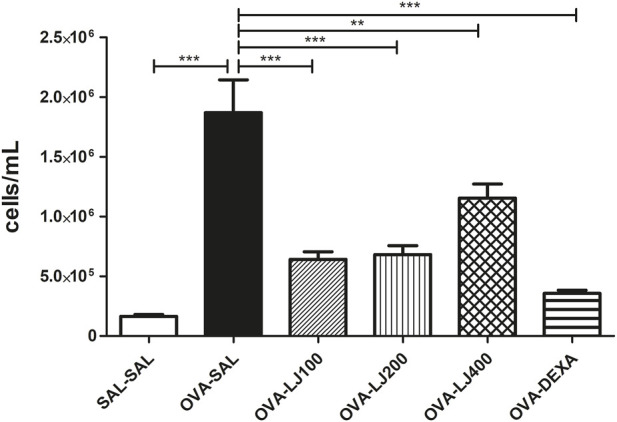
Effects of a hydro-alcoholic extract of *Leonurus japonicus* on the total cell count in the bronchoalveolar lavage of ovalbumin (OVA)-challenged mice. OVA-SAL: sensitized and challenged with OVA and treated with saline solution; SAL-SAL: sensitized, challenged and treated with saline solution; OVA-LJ100, OVA-LJ200, OVA- LJ400: sensitized and challenged with OVA and treated with 100, 200 and 400 mg/kg of LJ, respectively; OVA-DEXA: sensitized and challenged with OVA and treated with 2 mg/kg dexamethasone. Values are shown as mean ± SEM (n = 8 per group). P < 0.001 (***).

**FIGURE 3:**
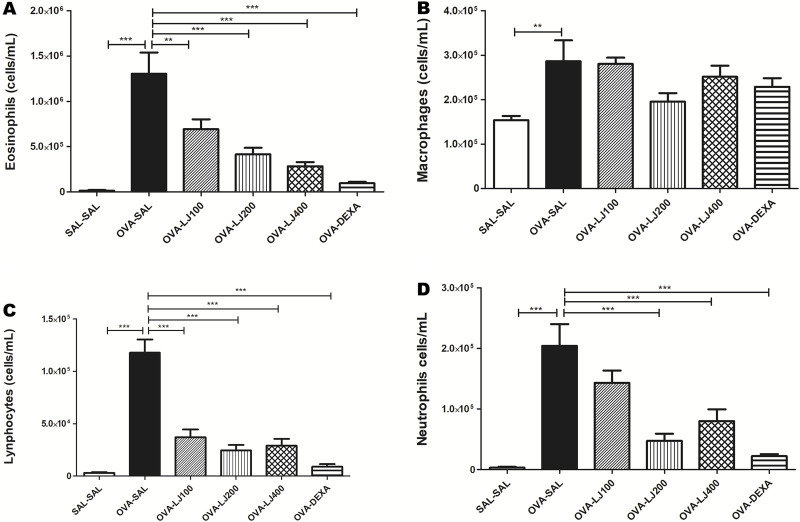
Effects of a hydro-alcoholic extract of *Leonurus japonicus* on the differential inflammatory cell count in the bronchoalveolar lavage of ovalbumin (OVA)-challenged mice. **(A)** eosinophils, **(B)** macrophages, **(C)** lymphocytes, and **(D)** neutrophils. OVA-SAL: sensitized and challenged with OVA and treated with saline solution; SAL-SAL: sensitized, challenged, and treated with saline solution; OVA-LJ100, OVA-S LJ200, OVA- LJ400: sensitized and challenged with OVA and treated with 100, 200 and 400 mg/kg of LJ, respectively; OVA-DEXA: sensitized and challenged with OVA and treated with 2 mg/kg dexamethasone. Values are shown as mean ± SEM (n = 8 per group). P < 0.01 (**) and P < 0.001 (***).

Total and differential cell counts in BAL are shown in Figures 2, 3. Total cell counts, as well as eosinophil, macrophage, lymphocyte, and neutrophil numbers, were significantly increased in OVA-challenged mice compared to saline-challenged mice. Treatment with *L. japonicus* at all doses and dexamethasone significantly reduced total cell (Figure 2), eosinophilic (Figure 3A), and lymphocytes (Figure 3C) counts in BAL compared to OVA-SAL group. Neutrophil numbers were significantly decreased only in the OVA-LJ200, OVA-LJ400, and OVA-DEXA groups compared to the OVA-SAL group (Figure 3D).

### 3.3 Modulation of lung cytokines and serum IgE levels

**FIGURE 4:**
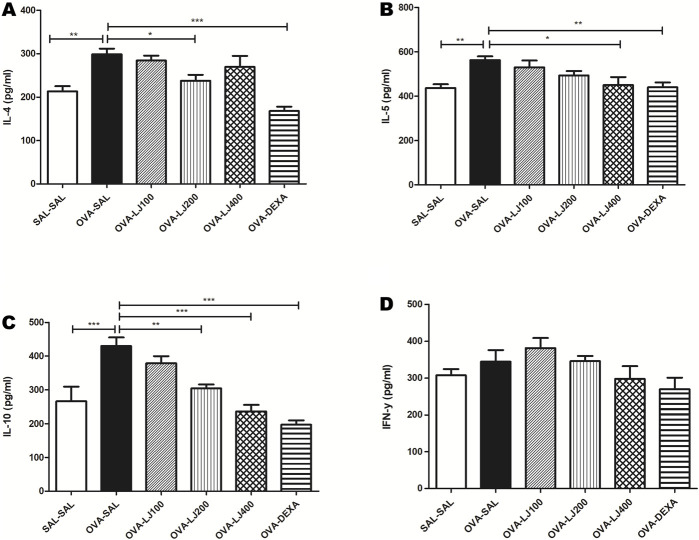
Effects of a hydro-alcoholic extract of *Leonurus japonicus* on the levels of cytokines in lung tissue of ovalbumin (OVA)-challenged mice. **(A)** IL-4, **(B)** IL-5, **(C)** IL-10, and **(D)** IFN-Ƴ. OVA-SAL: sensitized and challenged with OVA and treated with saline solution; SAL-SAL: sensitized, challenged, and treated with saline solution; OVA-LJ100, OVA- LJ200, OVA- LJ400: sensitized and challenged with OVA and treated with 100, 200 and 400 mg/kg of LJ, respectively; OVA-DEXA: sensitized and challenged with OVA and treated with 2 mg/kg dexamethasone. Values are shown as mean ± SEM (n = 8 per group). P < 0.05 (*), P < 0.01 (**) and P < 0.001 (***).

**FIGURE 5:**
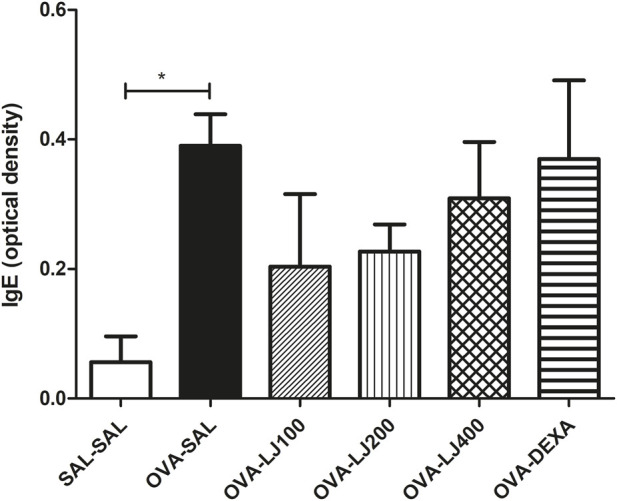
Effects of a hydro-alcoholic extract of *Leonurus japonicus* on the levels of OVA-specific IgE in the blood of ovalbumin (OVA)-challenged mice. OVA-SAL: sensitized and challenged with OVA and treated with saline solution; SAL-SAL: sensitized, challenged, and treated with saline solution; OVA-LJ100, OVA-LJ200, OVA- LJ400: sensitized and challenged with OVA and treated with 100, 200 and 400 mg/kg of LJ, respectively; OVA-DEXA: sensitized and challenged with OVA and treated with 2 mg/kg dexamethasone. Values are shown as mean ± SEM (n = 8 per group). P < 0.05 (*).

The levels of IL-4, IL-5, and IL-10 were significantly increased in OVA-challenged mice compared with saline-challenged mice. Treatment with *L. japonicus* modulated Th2 cytokine production in lung tissue, particularly IL-4, IL-5, and IL-10, without significantly altering IFN-γ or OVA-specific IgE levels (Figures 4, 5). Administration of 100 mg/kg of *L. japonicus* did not significantly change cytokine levels (Figure 4), whereas the 200 mg/kg dose significantly reduced IL-4 (Figure 4A) and IL-10 (Figure 4C) levels, and the 400 mg/kg dose significantly decreased IL-5 (Figure 4B) and IL-10 (Figure 4C) levels compared to the OVA-SAL group. Dexamethasone significantly reduced IL-4, IL-5, and IL-10 levels compared to OVA-challenged mice. Serum levels of OVA-specific IgE were not significantly altered by any treatment (Figure 5).

### 3.4 Effect of *L. japonicus* on peribronchial inflammation and mucus production

**FIGURE 6:**
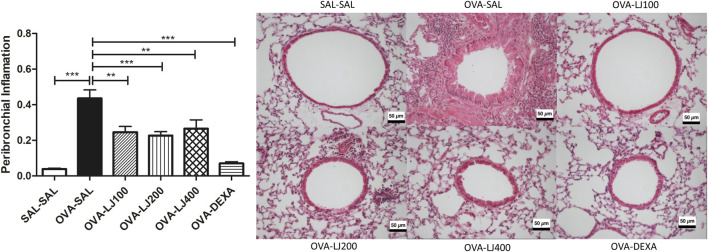
Effects of a hydro-alcoholic extract of *Leonurus japonicus* on the levels of peribronchial inflammation of ovalbumin (OVA)-challenged mice. Assessment of peribronchial inflammation followed the methodology of Weibel (2010). OVA-SAL: sensitized and challenged with OVA and treated with saline solution; SAL-SAL: sensitized, challenged, and treated with saline solution; OVA-LJ100, OVA-LJ200, OVA-LJ400: sensitized and challenged with OVA and treated with 100, 200, 400 mg/kg of LJ, respectively; OVA-DEXA: sensitized and challenged with OVA and treated with 2 mg/kg dexamethasone. Values are shown as mean ± SEM (n = 8 per group). P < 0.05 (*), P < 0.01 (**) and P < 0.001 (***). The histopathological images (hematoxylin-eosin stain; ×200 magnification) show inflammatory cells in lung tissue of all groups.

**FIGURE 7:**
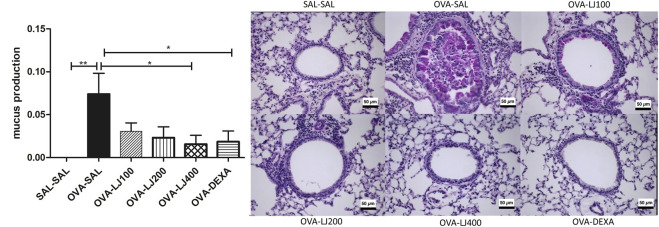
Effects of a hydro-alcoholic extract of *Leonurus japonicus* on the levels of mucus production of ovalbumin (OVA)-challenged mice. Assessment of mucus production followed the methodology of Weibel (2010). OVA-SAL: sensitized and challenged with OVA and treated with saline solution; SAL-SAL: sensitized, challenged, and treated with saline solution; OVA-LJ100, OVA-LJ200, OVA-LJ400: sensitized and challenged with OVA and treated with 100, 200, 400 mg/kg of LJ, respectively; OVA-DEXA: sensitized and challenged with OVA and treated with 2 mg/kg dexamethasone. Values are shown as mean ± SEM (n = 8 per group). P < 0.05 (*), P < 0.01 (**) and P < 0.001 (***). The histopathological images (periodic acid-Schiff stain; ×200 magnification) show mucus production in lung tissue of all groups.

OVA-challenged mice exhibited a significant increase in peribronchial inflammation and mucus production compared to saline-challenged controls (Figures 6, 7). Treatment with *L. japonicus* extract at all doses (100, 200, and 400 mg/kg), as well as dexamethasone, significantly reduced peribronchial inflammation in lung tissue (Figure 6), whereas the 400 mg/kg dose and dexamethasone significantly reduced mucus production (Figure 7).

### 3.5 Phytochemical composition of *L. japonicus* extract

**FIGURE 8:**
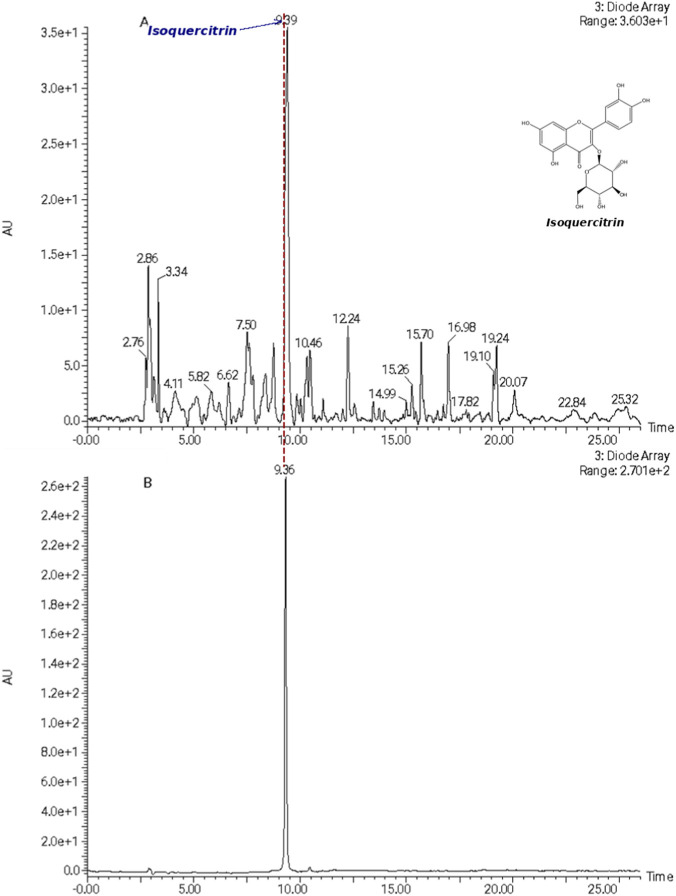
Ultra performance liquid chromatograms of the hydro-alcoholic extract of *Leonurus japonicus* **(A)** of the standard compound isoquercitrin **(B)**.

LC-MS/MS analysis identified isoquercitrin as the major compound in the *L. japonicus* extract, along with other phenolic constituents (Table 1; Figure 8). Thirteen compounds were putatively identified in the *L. japonicus* extract. Their retention times, elemental formulas, experimental m/z values, and major MS/MS fragments are summarized in Table 1. These compounds belonged primarily to three major chemical classes: flavonol glycosides (including rutin, isoquercitrin, and several kaempferol derivatives), phenolic acids (such as chlorogenic acid, sinapic acid, and 3,4-di-O-caffeoylquinic acid), and an amino acid derivative (N-acetyltryptophan). These metabolites are widely recognized for their antioxidant and anti-inflammatory properties, which may contribute to the extract’s observed biological activity.

**TABLE 1:** Annotated compounds in *Leonurus japonicus* hydro-alcoholic extract by high-performance liquid chromatography–quadrupole-time of flight mass spectrometry (HPLC–QTOF-MS).

Rt (min)	Exp. m/z [M-H]-	Mass accuracy (mDa)	Molecular formula	Annotated compound	MS/MS fragments*	References
3.86	353.0868	0.58	C_16_H_18_O_9_	Chlorogenic acid	191.0553, 127.0388, 85.0281	Horai et al. (2010); Vaniya and Fiehn (2015); Zhang et al. (2023)
3.89	163.0403	0.26	C_9_H_8_O_3_	3-Hydroxycinnamic acid	163.0408, 119.0479, 91.0577, 71.0226	Horai et al. (2010); Vaniya and Fiehn (2015); Zhang et al. (2023)
5.44	625.1398	1.22	C_27_H_30_O_17_	Quercetin-3-O-sophoroside	625.4420, 463.0967, 300.0220, 197.0408	Horai et al. (2010); Vaniya and Fiehn (2015); Lee et al. (2020); Wei et al. (2023); Zhang et al. (2023)
5.96	609.1412	4.95	C_27_H_30_O_16_	Quercetin-3-O-rutinoside; Rutin	609.1423, 300.0128, 271.0202, 255.0265, 151.0006	Horai et al. (2010); Vaniya and Fiehn (2015); Lee et al. (2020); Wei et al. (2023); Zhang et al. (2023)
6.26	463.0879	0.52	C_21_H_20_O_12_	Quercetin-3-O-glucoside	463.0853,300.0279, 271.0251, 255.0312, 178.9957, 150.9999, 125.0181	Horai et al. (2010); Vaniya and Fiehn (2015); Lee et al. (2020); Wei et al. (2023); Zhang et al. (2023)
6.32	577.1545	1.83	C_27_H_30_O_14_	Kaempferol 3,7-dirhamnoside	577.1470, 430.0912, 285.0329	Horai et al. (2010); Vaniya and Fiehn (2015); Lee et al. (2020); Wei et al. (2023); Zhang et al. (2023)
6.43	245.0907	2.41	C_13_H_14_N_2_O_3_	N-Acetyltryptophan	245.0930, 203.0987, 116.0378	Horai et al. (2010); Vaniya and Fiehn (2015)
6.49	223.0627	1.48	C_11_H_12_O_5_	Sinapic acid	223.0626, 208.0424, 193.0072, 164.0424,149.0233, 135.1792, 121.0315	Horai et al. (2010); Vaniya and Fiehn (2015); Zhang et al. (2023)
6.52	593.1482	2.99	C_27_H_30_O_15_	Kaempferol-3-O-rutinoside	593.1501, 285.0404, 255.0287, 227.0310	Horai et al. (2010); Vaniya and Fiehn (2015); Lee et al. (2020); Wei et al. (2023); Zhang et al. (2023)
6.56	515.1214	2.20	C_25_H_24_O_12_	3,4-di-O-Caffeoylquinic acid	515.1138, 353.0893, 335.0767, 203.0351, 191.0535, 179.0358, 173.0488	Horai et al. (2010); Vaniya and Fiehn (2015); Zhang et al. (2023)
6.81	447.0947	1.41	C_21_H_20_O_11_	Kaempferol-3-O-glucoside	447.0943, 327.0754, 285.0428, 255.0315, 227.0293, 151.0027	Horai et al. (2010); Vaniya and Fiehn (2015); Lee et al. (2020); Wei et al. (2023); Zhang et al. (2023)
8.48	593.1318	1.77	C_30_H_26_O_13_	Kaempferol-3-O-glucoside-6″-p-coumaroyl	593.1328, 447.0958, 307.0881, 285.0369, 255.0328, 227.0368, 145.0283	Horai et al. (2010); Vaniya and Fiehn (2015); Lee et al. (2020); Wei et al. (2023); Zhang et al. (2023)
11.27	299.0588	2.72	C_16_H_12_O_6_	Kaempferol 3-methyl ether	299.0556, 284.0305, 255.0294, 227.0244	Horai et al. (2010); Vaniya and Fiehn (2015); Lee et al. (2020); Wei et al. (2023); Zhang et al. (2023)

Footnote. Compounds in this table are reported as annotated metabolites according to the four-level confidence framework proposed by Schymanski et al. (2014). Only quercetin-3-O-glucoside/isoquercitrin (compound 5) was confirmed at Schymanski Level 1 (confirmed structure) by co-injection with an authentic commercial standard using an Ascentis Express C18 column coupled to a Xevo TQ-S, triple-quadrupole mass spectrometer with diode array detection (Figure 8), and by ^1^H/^13^C NMR, analysis (Supplementary Material, Section B). The remaining twelve compounds were annotated at Schymanski Level 2 (probable structure), based on accurate-mass [M−H]^-^ data (mass accuracy ≤5 mDa), MS/MS, fragmentation patterns matched against the MoNA “MSMS-Public-Neg-VS11. msp” database (Horai et al., 2010) and the Vaniya–Fiehn Natural Products Library (Vaniya and Fiehn, 2015), data processing using MS-DIAL (Tsugawa et al., 2015) and MS-FINDER (Tsugawa et al., 2016), and consistency with previously reported phytochemical profiles of *Leonurus* spp. (Lee et al., 2020; Wei et al., 2023; Zhang et al., 2023). The corresponding MS/MS, spectra are provided in Supplementary Material, Section A (Supplementary Figures S2-S14).

* MS/MS, fragments refer to the major product ions observed in negative ESI, mode.

Isoquercitrin, identified as the major compound (Figure 8), was further isolated and structurally confirmed by ^1^H and ^13^C NMR. Detailed spectral data are provided in the Supplementary Material (Section B).

## 4 Discussion

The hydro-alcoholic extract of L*. japonicus* significantly improved asthma-like features in an experimental model. Its administration reduced BHR, influx of inflammatory cell into BAL, lung inflammation, mucus production, and the levels of key cytokines (IL-4, IL-5, and IL-10) in lung homogenates.

IL-4 and IL-5 play central roles in the pathogenesis of asthma. IL-4 promotes Th2 differentiation and IgE production, while IL-5 is essential for eosinophil recruitment and activation (Hammad and Lambrecht, 2021). The reduction of these cytokines in *L. japonicus*–treated animals may explain the observed decrease in eosinophil and lymphocyte counts in BAL.

Although IL-10 is typically produced as a compensatory mechanism to control excessive inflammation (Böhm et al., 2015), its reduction in this model may reflect decreased inflammatory stimulus, reducing the need for counter-regulation. Similar reductions in IL-10 levels have been reported in other preclinical models following anti-inflammatory treatment (Amorim et al., 2018; Li et al., 2021). Additionally, the extract may have contributed to a more regulated pulmonary environment by limiting the activation of IL-10-producing cells.

Previous studies have highlighted the anti-inflammatory effects of *L. japonicus* (Wei et al., 2023; Zhang et al., 2023; Shin et al., 2024), suggesting that these effects may be linked to the plant’s ability to modulate the production and release of key pro-inflammatory mediators, such as ROS (Shin et al., 2024), TNF-α, IL-6, and IL-8, potentially through inhibition of the NF-κB signaling pathway (Zhang et al., 2023; Shin et al., 2024).

Among the metabolites identified in the hydro-alcoholic extract of *L. japonicus* (Table 1) are sinapic acid, chlorogenic acid, and rutin, all of which have documented anti-inflammatory and anti-asthmatic activities. Sinapic acid has been shown to reduce pulmonary inflammation and Th2 cytokine levels (IL-4, IL-5, and IL-13), as well as serum IgE concentrations in a murine model of allergic asthma (Saeedavi et al., 2022). Similarly, chlorogenic acid suppressed pulmonary eosinophilia, IgE production, and inhibited activation of the STAT-6 and JNK signaling (Kim et al., 2010). Rutin has also been reported to reduce airway resistance and the recruitment of inflammatory cells in OVA-sensitized guinea pigs (Jung et al., 2007). The presence of these compounds in our extract supports its potential anti-asthmatic activity and is consistent with the pharmacological mechanisms previously described in the literature.

Isoquercitrin, identified as the major compound in the *L. japonicus* extract, is a flavonoid glycoside with well-documented preclinical anti-inflammatory and bronchoprotective properties. It exerts anti-inflammatory effects by targeting key signaling pathways. In LPS-stimulated macrophages, isoquercitrin inhibits NF-κB signaling, reduces nitric oxide (NO) and prostaglandin E2 (PGE2) production, and suppresses pro-inflammatory cytokine expressions (Lee et al., 2022). Isoquercitrin has also been shown to reduce osteoclast-driven bone loss in arthritis models through the Nrf2/ROS/NF-κB axis (Liu et al., 2024), and to suppresses NLRP3 inflammasome activation by downregulating HSP90, caspase-1, and IL-1β (Ma et al., 2023). In various *in vivo* preclinical models, isoquercitrin reduces eosinophil infiltration, suppress IL-5 levels production (Rogerio et al., 2007), and exhibits bronchodilator effect (Fernandez et al., 2005). Given these mechanisms, the reductions in eosinophils, lymphocytes, and neutrophils, the improvement in lung mechanics, and the decreased levels of Th2 cytokines observed in our study are consistent with previous findings and may be associated with NF-κB inhibition and subsequent downregulation of inflammatory gene expression.

Isoquercitrin has demonstrated additional anti-inflammatory effects, including histamine inhibition, suppression of Th2 cytokines (Bui et al., 2019), and blockade of the MAPK and NF-κB signaling pathways (Li et al., 2016; Lee et al., 2022).

Regarding safety, the literature has shown that *Leonurus* spp. presents a favorable safety profile at standard doses. Toxicity concerns, primarily hepatic and renal, are confined to high or excessive doses and appear to be dependent on the extraction method and specific compounds, with alkaloids identified as the main toxic constituents. Proposed mechanisms include oxidative stress and increased cell apoptosis in renal tissue. Importantly, no significant organ damage has been observed at therapeutic doses, and no serious adverse reactions have been reported in clinical use (Wang et al., 2025). In our study, no apparent signs of toxicity were observed under the experimental conditions evaluated.

Limitations of this study include: (a) the effects of isolated isoquercitrin and others metabolites were not directly investigated; therefore, the observed effects likely reflect a combined (possibly synergistic) action of multiple constituents, although prior evidence supports the anti-inflammatory effects of isoquercitrin; (b) the minimal effective dose of the extract was not determined; (c) IL-13 and epithelial alarmins (TSLP, IL-33, and IL-25) were not measured and should be included in future studies to better characterize the underlying mechanisms; (d) the IP route used for treatment does not directly relate to the traditional oral route, but it ensures high bioavailability, which allows to detect the biological effect; (e) only male animals were studied, possibly introducing sex bias. Future studies should explore dose-response relationships and assess the specific effects of isolated compounds; (f) as an acute model of asthma was used, it was not possible to evaluate key components of airway remodeling (e.g., collagen deposition and smooth muscle mass); and (g) although the findings were consistent with other results, inflammation and mucus scoring was performed by a single unblinded scorer, and randomization and blinding were not implemented. Future studies should explore dose-response relationships and assess the specific effects of isolated compounds.

In conclusion, we demonstrated for the first time that a hydro-alcoholic extract of *L. japonicus* exerts significant anti-asthmatic effects in a murine model. Treatment improved pulmonary mechanics, reduced mucus production, decreased inflammation and modulated key cytokines involved in the allergic response. These effects may be associated with the combined activity of multiple bioactive compounds present in the extract, including isoquercitrin, identified as one of the principal constituents. Collectively, our preclinical findings support the traditional use of *L. japonicus* and highlight its potential as a source of therapeutic agents for allergic asthma. Further investigations are warranted to elucidate its mechanisms of action and assess its clinical relevance.

## Data Availability

The raw data supporting the conclusions of this article will be made available by the authors, without undue reservation.
