# Supplementary material for: Leonurus japonicus Houtt. extract containing isoquercitrin reduces airway inflammation in mice with allergic asthma
Source: Front Pharmacol. 2026 Jun 18;17:1813430. doi: 10.3389/fphar.2026.1813430 (PMC13322827; doi:10.3389/fphar.2026.1813430)
Supplement: Supplementary file 1 [file Supplementaryfile1.docx]

**Supplementary File**

**LC-QTOF-MS/MS chemical profiling of the *Leonurus japonicus* Houtt. hydro-alcoholic extract: Base peak chromatogram and MS/MS spectra of annotated compounds**

**Supplementary material associated with the manuscript:**
*Leonurus japonicus* Houtt. extract containing isoquercitrin reduces airway inflammation in mice with allergic asthma

**Analytical conditions.** Chemical profiling was performed on a Nexera X2 UHPLC system coupled to a TripleTOF 5600+ mass spectrometer (Sciex, Foster City, CA, USA) equipped with a Turbo-V Ion Spray source operating in negative mode (ESI−). Chromatographic separation was carried out on an Acquity XSelect HSS T3 column (2.1 × 150 mm, 2.5 µm, Waters), with mobile phase A (0.1 % v/v formic acid in water) and B (acetonitrile), under a linear gradient (0.0 min, 10 % B; 1–21 min, 90 % B; 21–25 min, 90 % B; 25.1–33.0 min, 10 % B). Identification of isoquercitrin was further confirmed on an Acquity UPLC H-Class system coupled to a Xevo TQ-S triple-quadrupole spectrometer (Waters), by co-injection with an authentic commercial standard.

**Identification level.** Compounds reported in Table 1 of the main manuscript are presented as annotated compounds, corresponding to Level 2 (probable structure) according to the metabolomics reporting standards of Schymanski et al. (Environ. Sci. Technol., 2014, 48, 2097–2098). Annotation was based on accurate-mass [M−H]⁻ (mass accuracy ≤ 5 mDa), MS/MS fragmentation pattern, and comparison with reference spectra and literature data. Specifically, compound annotation was performed in MS-DIAL (v. 4.7) coupled with MS-FINDER, using the public spectral library “MSMS-Public-Neg-VS11.msp” (MoNA), as described in section 2.3 of the main manuscript. Isoquercitrin was unambiguously confirmed (Level 1) by co-injection with an authentic standard and by ¹H/¹³C NMR (Supplementary File – Section B).

**Data availability.** Raw LC-MS/MS data (.wiff/.mzML) and the NMR free induction decay files for isoquercitrin have been deposited in the Open Science Framework (OSF) repository, available at https://osf.io/zh3p5/.

**Overview of supplementary figures**

| **Figure** | **Panel / compound no.** | **Compound / content** | **RT (min)** | **Exp. m/z [M−H]⁻** | **Major MS/MS fragments / description** |
| --- | --- | --- | --- | --- | --- |
| S1 | Base peak chromatogram (BPC) | *Leonurus japonicus* hydro-alcoholic extract | - | - | Numbered peaks correspond to annotated compounds listed in Table 1 and to Figures S2-S14. |
| S2 | Compound 1 | Chlorogenic acid | 3.86 | 353.0868 | 191.0553, 127.0388, 85.0281 |
| S3 | Compound 2 | 3-Hydroxycinnamic acid | 3.89 | 163.0403 | 163.0408, 119.0479, 91.0577, 71.0226 |
| S4 | Compound 3 | Quercetin 3-O-sophoroside | 5.44 | 625.1398 | 625.4420, 463.0967, 300.0220, 197.0408 |
| S5 | Compound 4 | Quercetin-3-O-rutinoside (Rutin) | 5.96 | 609.1412 | 609.1423, 300.0128, 271.0202, 255.0265, 151.0006 |
| S6 | Compound 5 | Quercetin-3-O-glucoside (Isoquercitrin) | 6.26 | 463.0879 | 463.0853, 300.0279, 271.0251, 255.0312, 178.9957, 150.9999, 125.0181 |
| S7 | Compound 6 | Kaempferol 3,7-dirhamnoside | 6.32 | 577.1545 | 577.1470, 430.0912, 285.0329 |
| S8 | Compound 7 | N-Acetyltryptophan | 6.43 | 245.0907 | 245.0930, 203.0987, 116.0378 |
| S9 | Compound 8 | Sinapic acid | 6.49 | 223.0627 | 223.0626, 208.0424, 193.0072, 164.0424, 149.0233, 135.1792, 121.0315 |
| S10 | Compound 9 | Kaempferol-3-O-rutinoside (Nicotiflorin) | 6.52 | 593.1482 | 593.1501, 285.0404, 255.0287, 227.0310 |
| S11 | Compound 10 | 3,4-Di-O-caffeoylquinic acid | 6.56 | 515.1214 | 515.1138, 353.0893, 335.0767, 203.0351, 191.0535, 179.0358, 173.0488 |
| S12 | Compound 11 | Kaempferol-3-O-glucoside (Astragalin) | 6.81 | 447.0947 | 447.0943, 327.0754, 285.0428, 255.0315, 227.0293, 151.0027 |
| S13 | Compound 12 | Kaempferol-3-O-glucoside-6''-p-coumaroyl (Tiliroside) | 8.48 | 593.1318 | 593.1328, 447.0958, 307.0881, 285.0369, 255.0328, 227.0368, 145.0283 |
| S14 | Compound 13 | Kaempferol 3-methyl ether | 11.27 | 299.0588 | 299.0556, 284.0305, 255.0294, 227.0244 |

**Figure captions**

**Figure S1. Base peak chromatogram (BPC) of the *Leonurus japonicus* hydro-alcoholic extract.** BPC obtained on the Acquity XSelect HSS T3 column (2.1 × 150 mm, 2.5 µm) coupled to a TripleTOF 5600+ in ESI− mode. Numbered peaks correspond to the annotated compounds listed in Table 1 of the main manuscript and detailed in Figures S2–S14.

**Figure S2. Compound 1 – Chlorogenic acid.** MS/MS spectrum acquired in ESI− mode at RT 3.86 min for [M−H]⁻ m/z 353.0868. Major annotated fragments: 191.0553, 127.0388, 85.0281.

**Figure S3. Compound 2 – 3-Hydroxycinnamic acid.** MS/MS spectrum acquired in ESI− mode at RT 3.89 min for [M−H]⁻ m/z 163.0403. Major annotated fragments: 163.0408, 119.0479, 91.0577, 71.0226.

**Figure S4. Compound 3 – Quercetin 3-O-sophoroside.** MS/MS spectrum acquired in ESI− mode at RT 5.44 min for [M−H]⁻ m/z 625.1398. Major annotated fragments: 625.4420, 463.0967, 300.0220, 197.0408.

**Figure S5. Compound 4 – Quercetin-3-O-rutinoside (Rutin).** MS/MS spectrum acquired in ESI− mode at RT 5.96 min for [M−H]⁻ m/z 609.1412. Major annotated fragments: 609.1423, 300.0128, 271.0202, 255.0265, 151.0006.

**Figure S6. Compound 5 – Quercetin-3-O-glucoside (Isoquercitrin).** MS/MS spectrum acquired in ESI− mode at RT 6.26 min for [M−H]⁻ m/z 463.0879. Major annotated fragments: 463.0853, 300.0279, 271.0251, 255.0312, 178.9957, 150.9999, 125.0181.

**Figure S7. Compound 6 – Kaempferol 3,7-dirhamnoside.** MS/MS spectrum acquired in ESI− mode at RT 6.32 min for [M−H]⁻ m/z 577.1545. Major annotated fragments: 577.1470, 430.0912, 285.0329.

**Figure S8. Compound 7 – N-Acetyltryptophan.** MS/MS spectrum acquired in ESI− mode at RT 6.43 min for [M−H]⁻ m/z 245.0907. Major annotated fragments: 245.0930, 203.0987, 116.0378.

**Figure S9. Compound 8 – Sinapic acid.** MS/MS spectrum acquired in ESI− mode at RT 6.49 min for [M−H]⁻ m/z 223.0627. Major annotated fragments: 223.0626, 208.0424, 193.0072, 164.0424, 149.0233, 135.1792, 121.0315.

**Figure S10. Compound 9 – Kaempferol-3-O-rutinoside (Nicotiflorin).** MS/MS spectrum acquired in ESI− mode at RT 6.52 min for [M−H]⁻ m/z 593.1482. Major annotated fragments: 593.1501, 285.0404, 255.0287, 227.0310.

**Figure S11. Compound 10 – 3,4-Di-O-caffeoylquinic acid.** MS/MS spectrum acquired in ESI− mode at RT 6.56 min for [M−H]⁻ m/z 515.1214. Major annotated fragments: 515.1138, 353.0893, 335.0767, 203.0351, 191.0535, 179.0358, 173.0488.

**Figure S12. Compound 11 – Kaempferol-3-O-glucoside (Astragalin).** MS/MS spectrum acquired in ESI− mode at RT 6.81 min for [M−H]⁻ m/z 447.0947. Major annotated fragments: 447.0943, 327.0754, 285.0428, 255.0315, 227.0293, 151.0027.

**Figure S13. Compound 12 – Kaempferol-3-O-glucoside-6''-p-coumaroyl (Tiliroside).** MS/MS spectrum acquired in ESI− mode at RT 8.48 min for [M−H]⁻ m/z 593.1318. Major annotated fragments: 593.1328, 447.0958, 307.0881, 285.0369, 255.0328, 227.0368, 145.0283.

**Figure S14. Compound 13 – Kaempferol 3-methyl ether.** MS/MS spectrum acquired in ESI− mode at RT 11.27 min for [M−H]⁻ m/z 299.0588. Major annotated fragments: 299.0556, 284.0305, 255.0294, 227.0244.

━━━━━━━━━━━━━━━━━━━━━━━━━━━━━━━━━━━━━━━━━━

**Section A - LC-QTOF-MS/MS SECTION**

*Base peak chromatogram and MS/MS spectra of annotated compounds*

**Figures S1 – S14**

━━━━━━━━━━━━━━━━━━━━━━━━━━━━━━━━━━━━━━━━━━

*[Section A — LC-QTOF-MS/MS]*


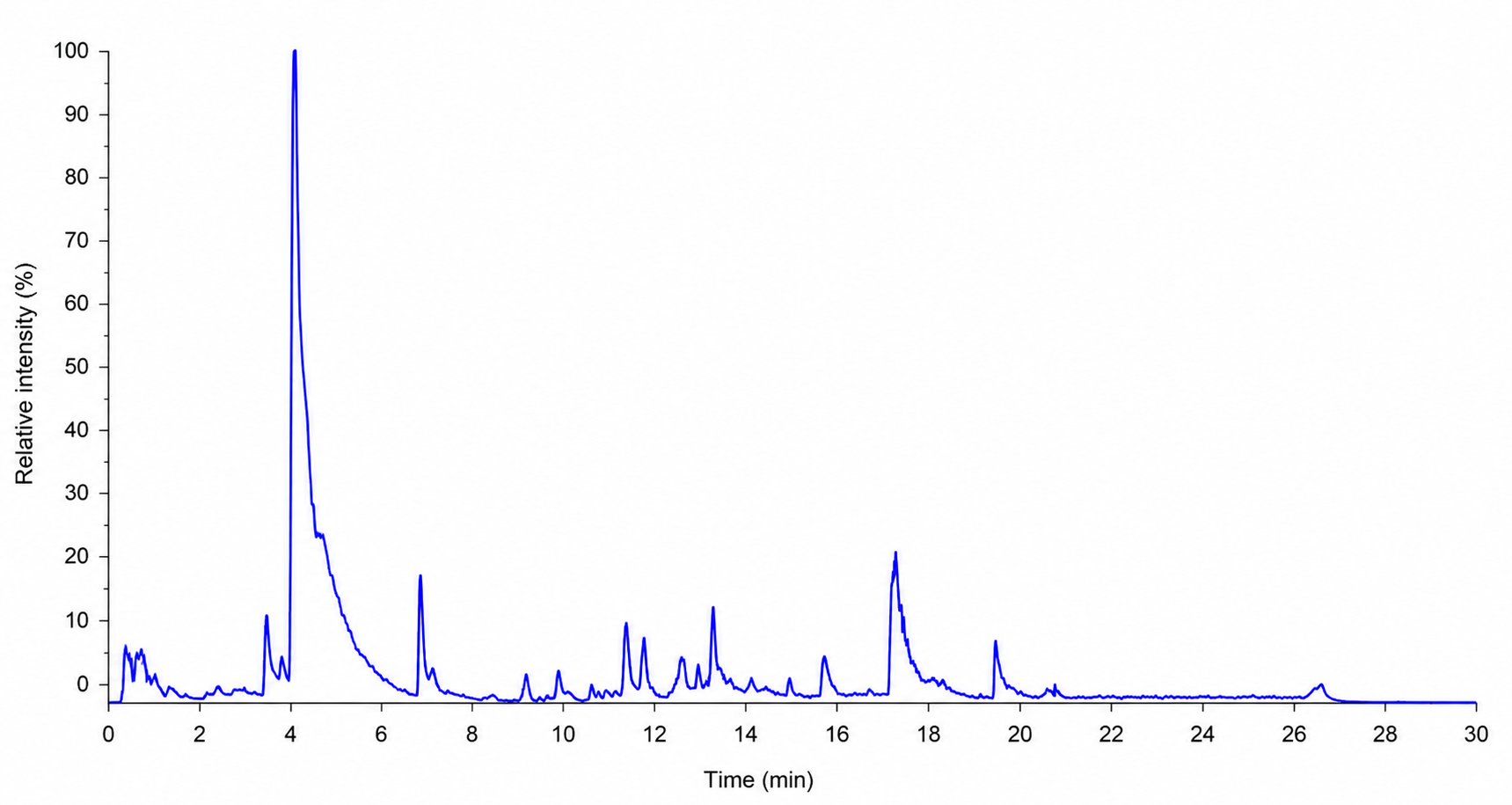


**Figure S1. *Base peak chromatogram (BPC) of the Leonurus japonicus hydro-alcoholic extract.*** BPC obtained on the Acquity XSelect HSS T3 column (2.1 × 150 mm, 2.5 µm) coupled to a TripleTOF 5600+ in ESI− mode.

*[Section A - LC-QTOF-MS/MS]*


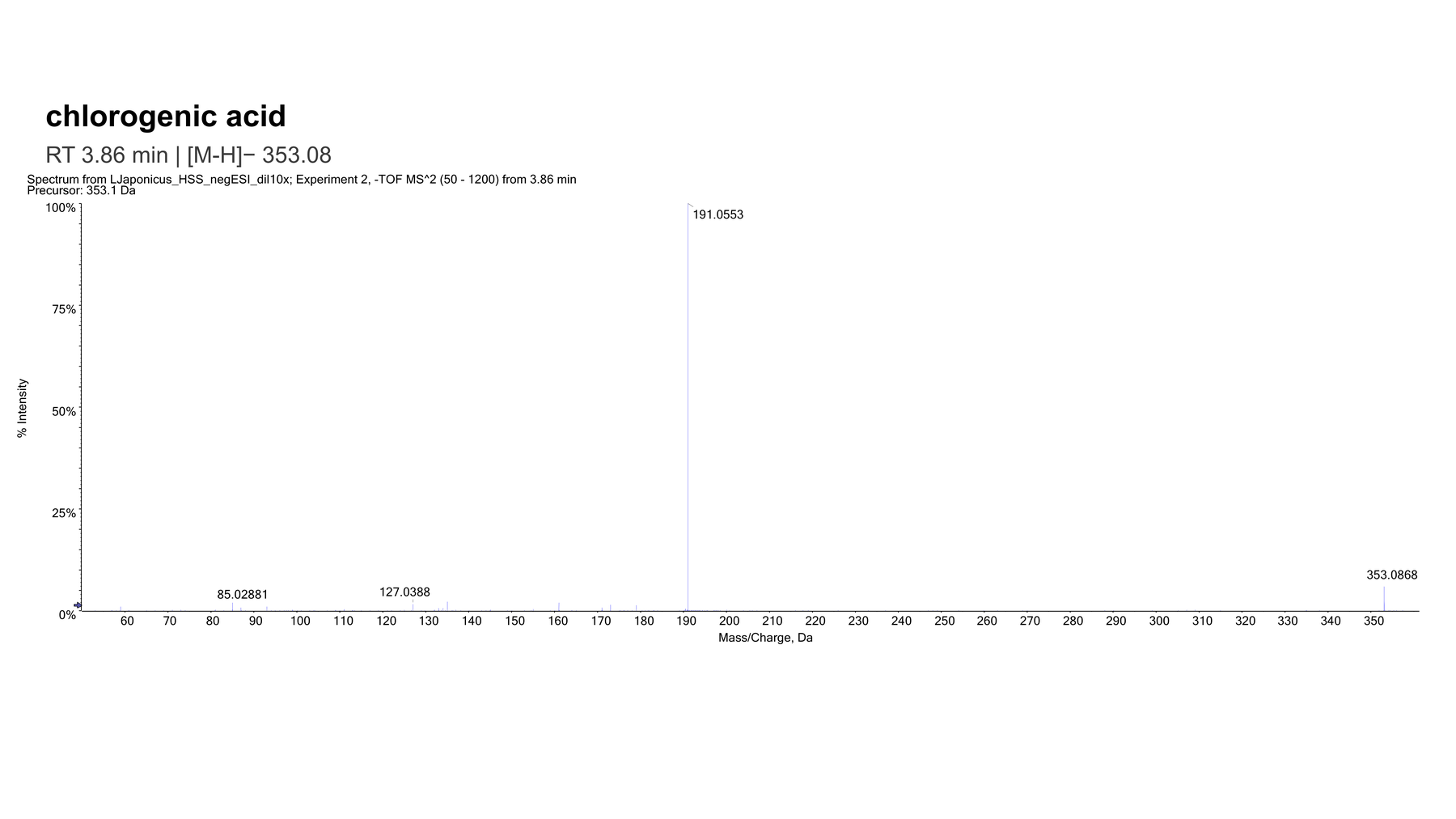


**Figure S2. *Compound 1 – Chlorogenic acid.*** MS/MS spectrum acquired in ESI− mode at RT 3.86 min for [M−H]⁻ m/z 353.0868. Major annotated fragments: 191.0553, 127.0388, 85.0281.

*[Section A - LC-QTOF-MS/MS]*


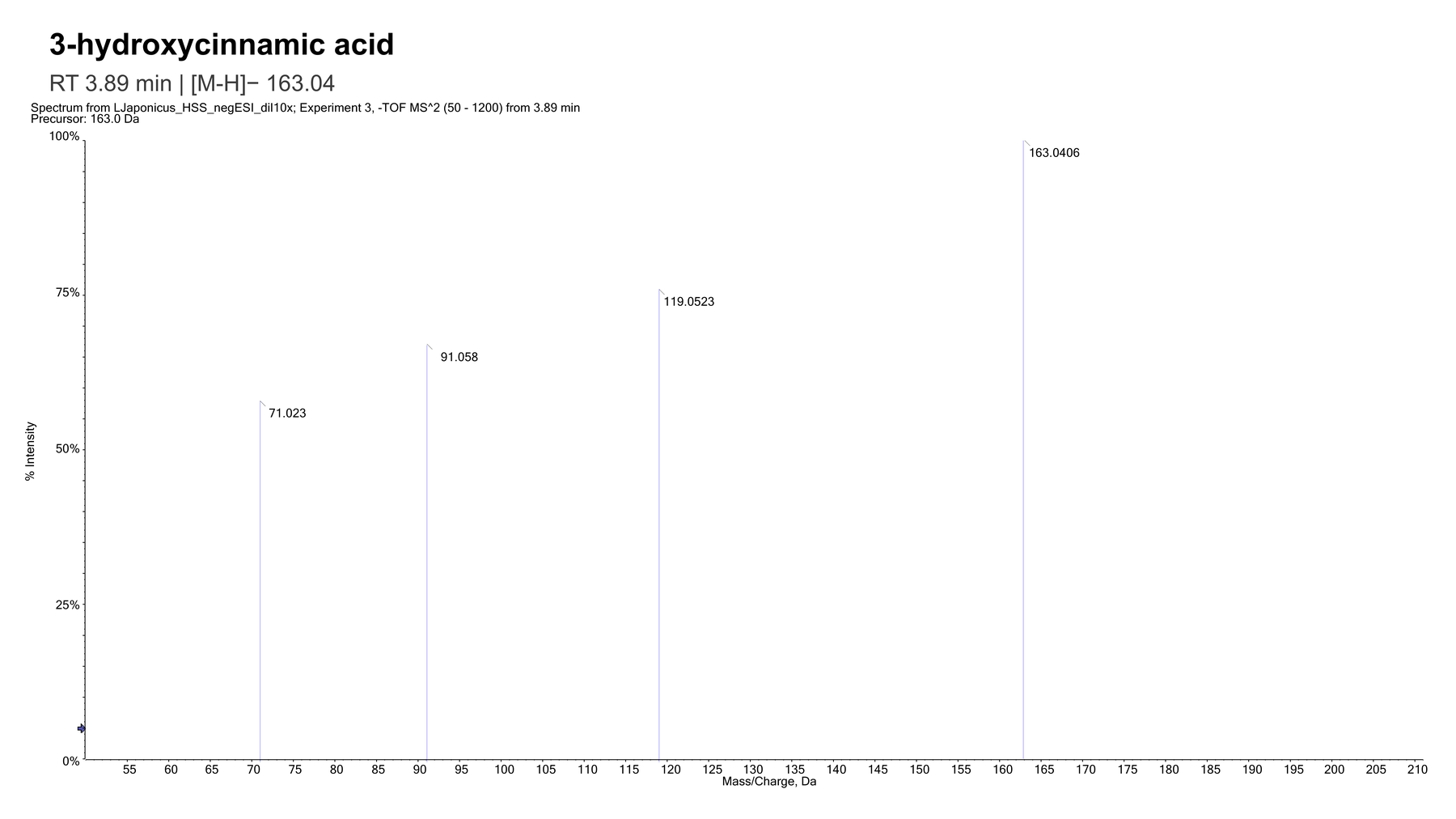


**Figure S3. *Compound 2 – 3-Hydroxycinnamic acid.*** MS/MS spectrum acquired in ESI− mode at RT 3.89 min for [M−H]⁻ m/z 163.0403. Major annotated fragments: 163.0408, 119.0479, 91.0577, 71.0226.

*[Section A - LC-QTOF-MS/MS]*


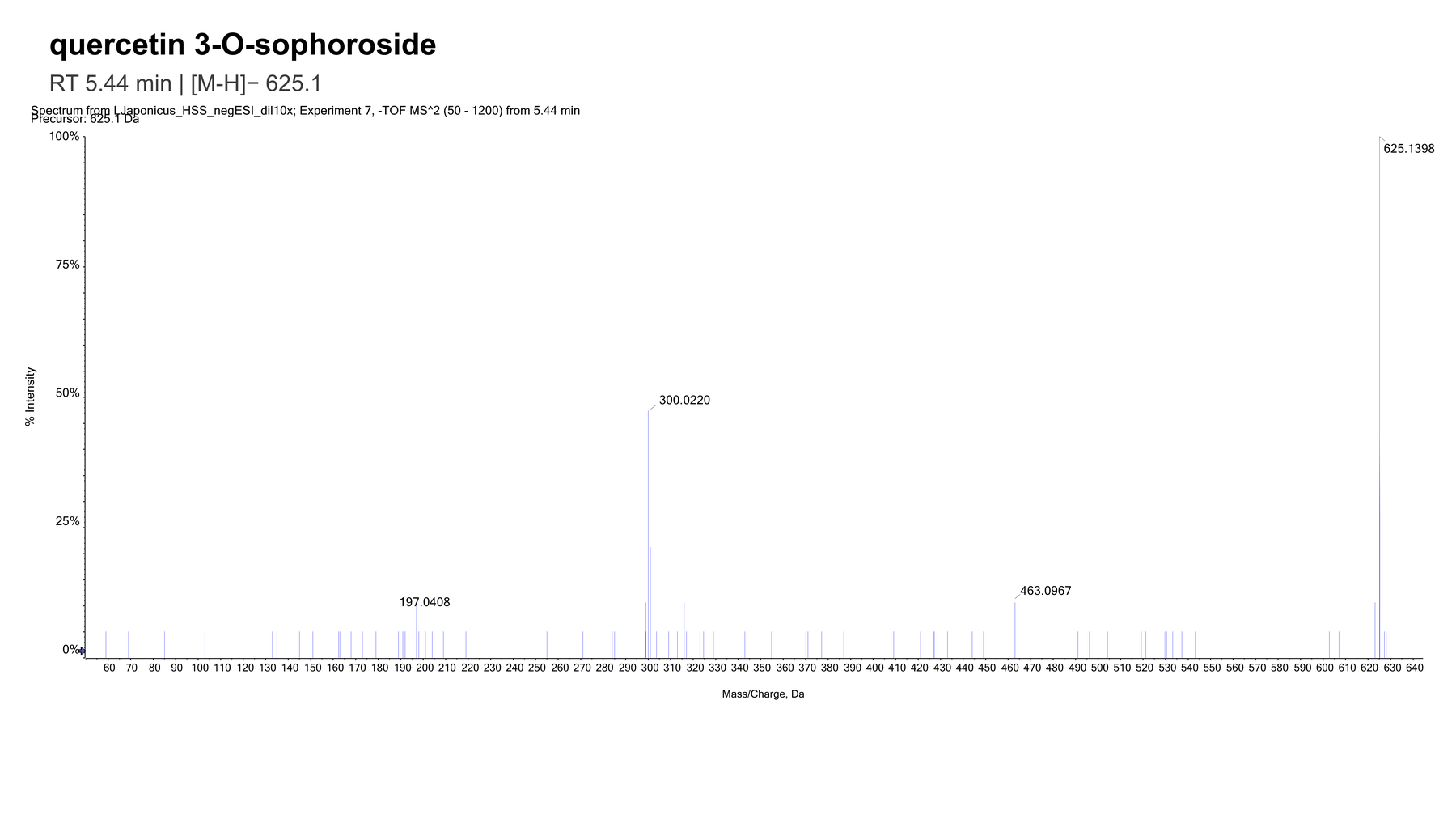


**Figure S4. *Compound 3 – Quercetin 3-O-sophoroside.*** MS/MS spectrum acquired in ESI− mode at RT 5.44 min for [M−H]⁻ m/z 625.1398. Major annotated fragments: 625.4420, 463.0967, 300.0220, 197.0408.

*[Section A - LC-QTOF-MS/MS]*


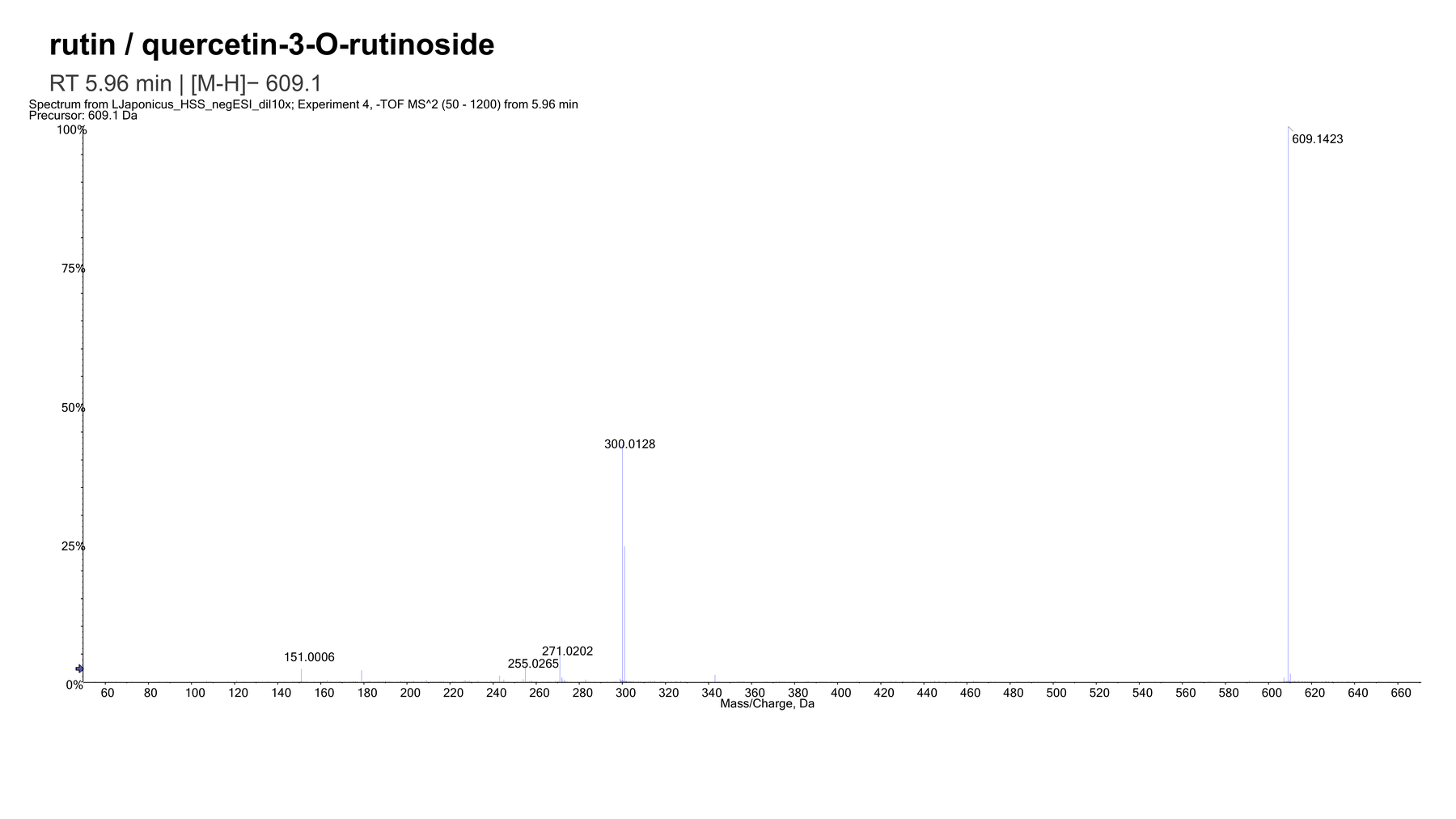


**Figure S5. *Compound 4 – Quercetin-3-O-rutinoside (Rutin).*** MS/MS spectrum acquired in ESI− mode at RT 5.96 min for [M−H]⁻ m/z 609.1412. Major annotated fragments: 609.1423, 300.0128, 271.0202, 255.0265, 151.0006.

*[Section A - LC-QTOF-MS/MS]*


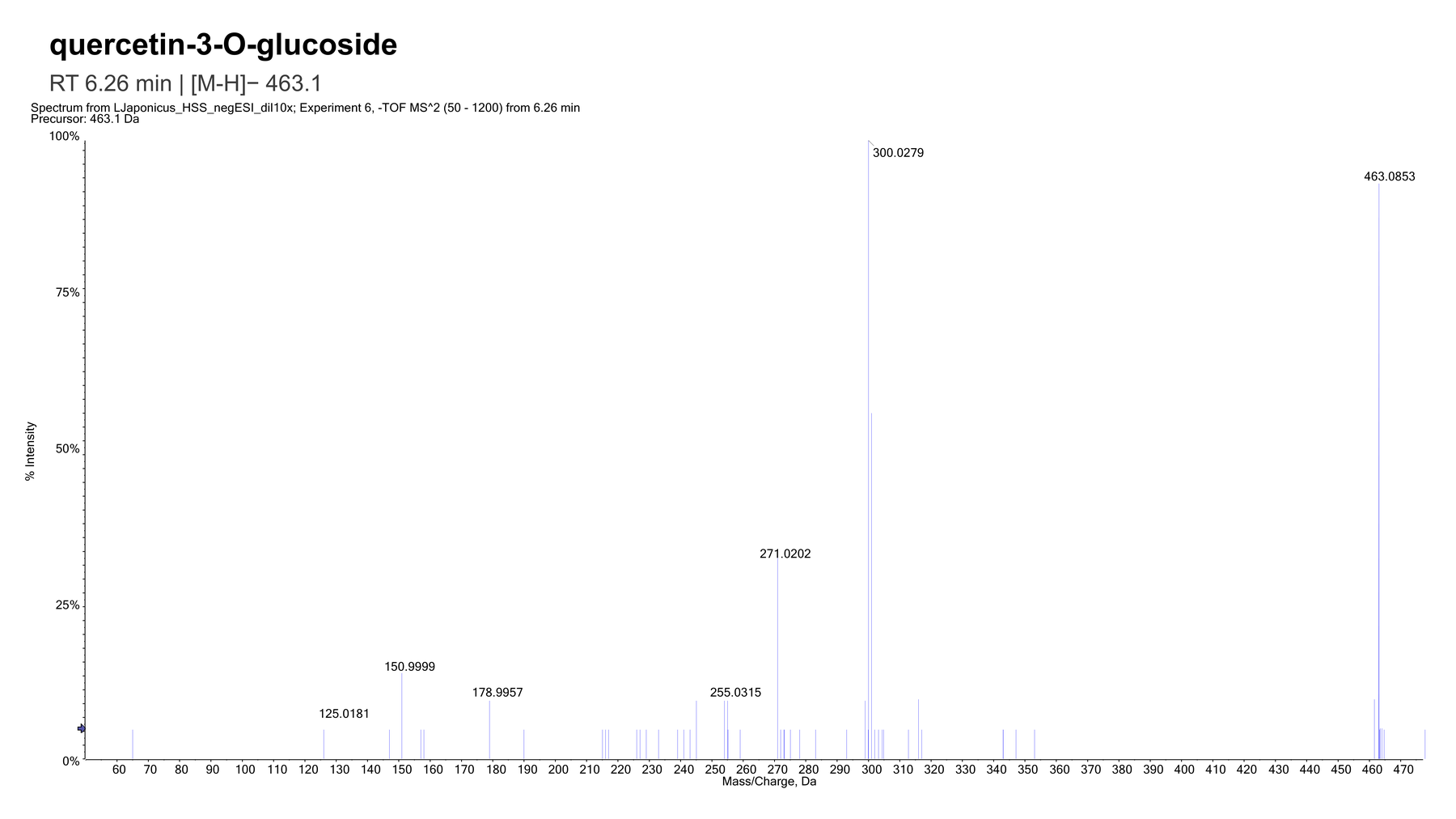


**Figure S6. *Compound 5 – Quercetin-3-O-glucoside (Isoquercitrin).*** MS/MS spectrum acquired in ESI− mode at RT 6.26 min for [M−H]⁻ m/z 463.0879. Major annotated fragments: 463.0853, 300.0279, 271.0251, 255.0312, 178.9957, 150.9999, 125.0181.

*[Section A - LC-QTOF-MS/MS]*


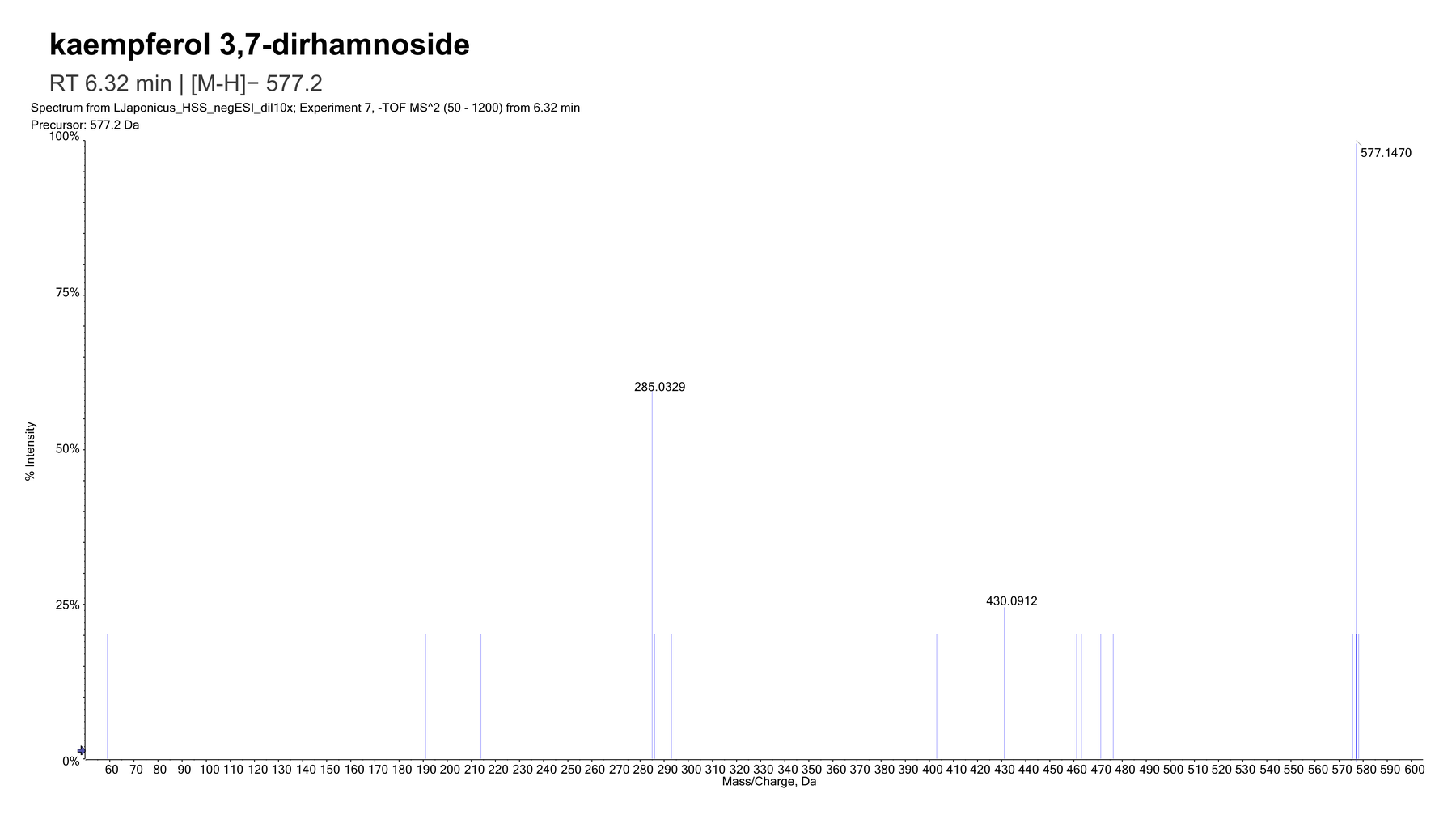


**Figure S7. *Compound 6 – Kaempferol 3,7-dirhamnoside.*** MS/MS spectrum acquired in ESI− mode at RT 6.32 min for [M−H]⁻ m/z 577.1545. Major annotated fragments: 577.1470, 430.0912, 285.0329.

*[Section A - LC-QTOF-MS/MS]*


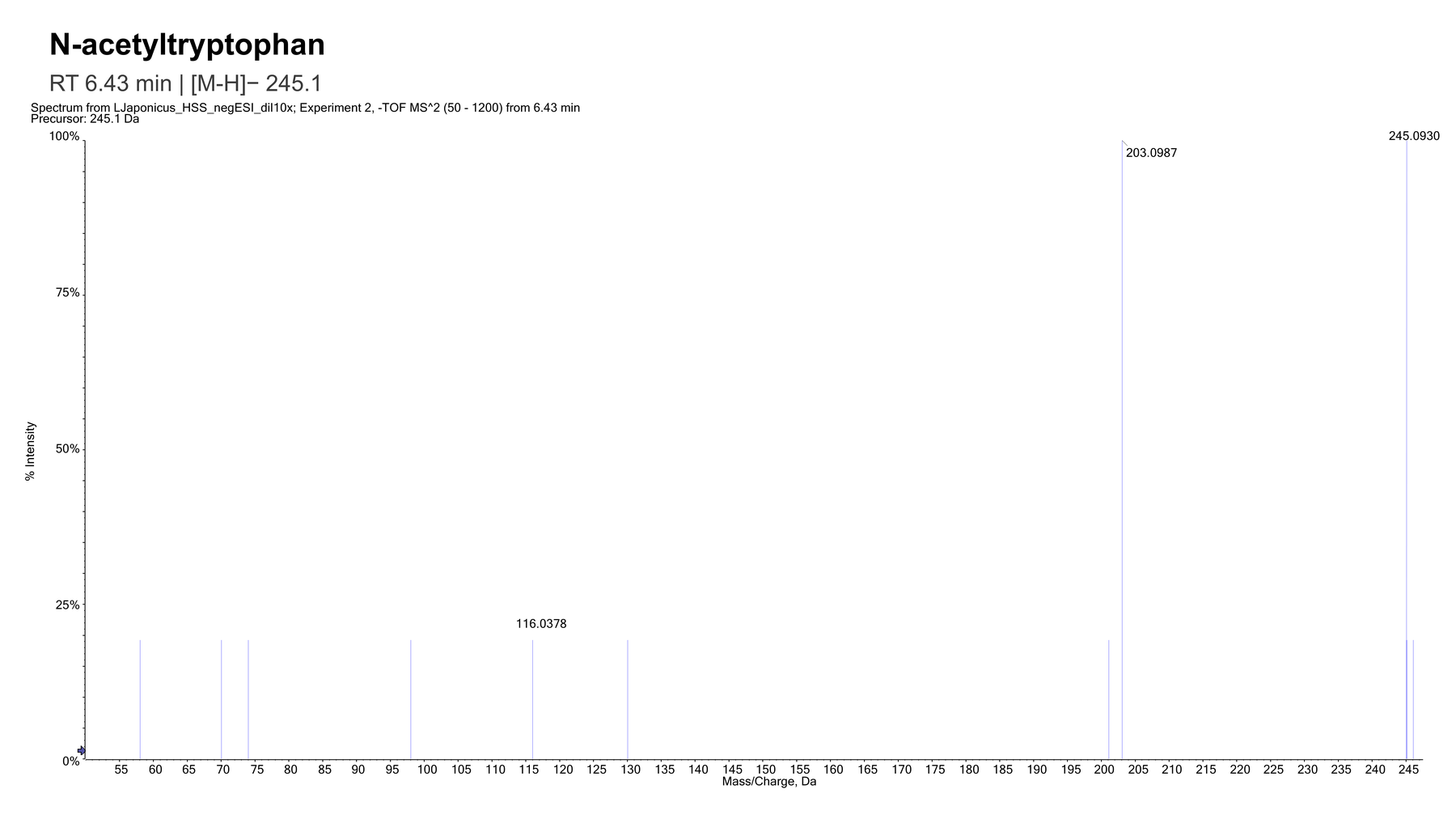


**Figure S8. *Compound 7 – N-Acetyltryptophan.*** MS/MS spectrum acquired in ESI− mode at RT 6.43 min for [M−H]⁻ m/z 245.0907. Major annotated fragments: 245.0930, 203.0987, 116.0378.

*[Section A - LC-QTOF-MS/MS]*


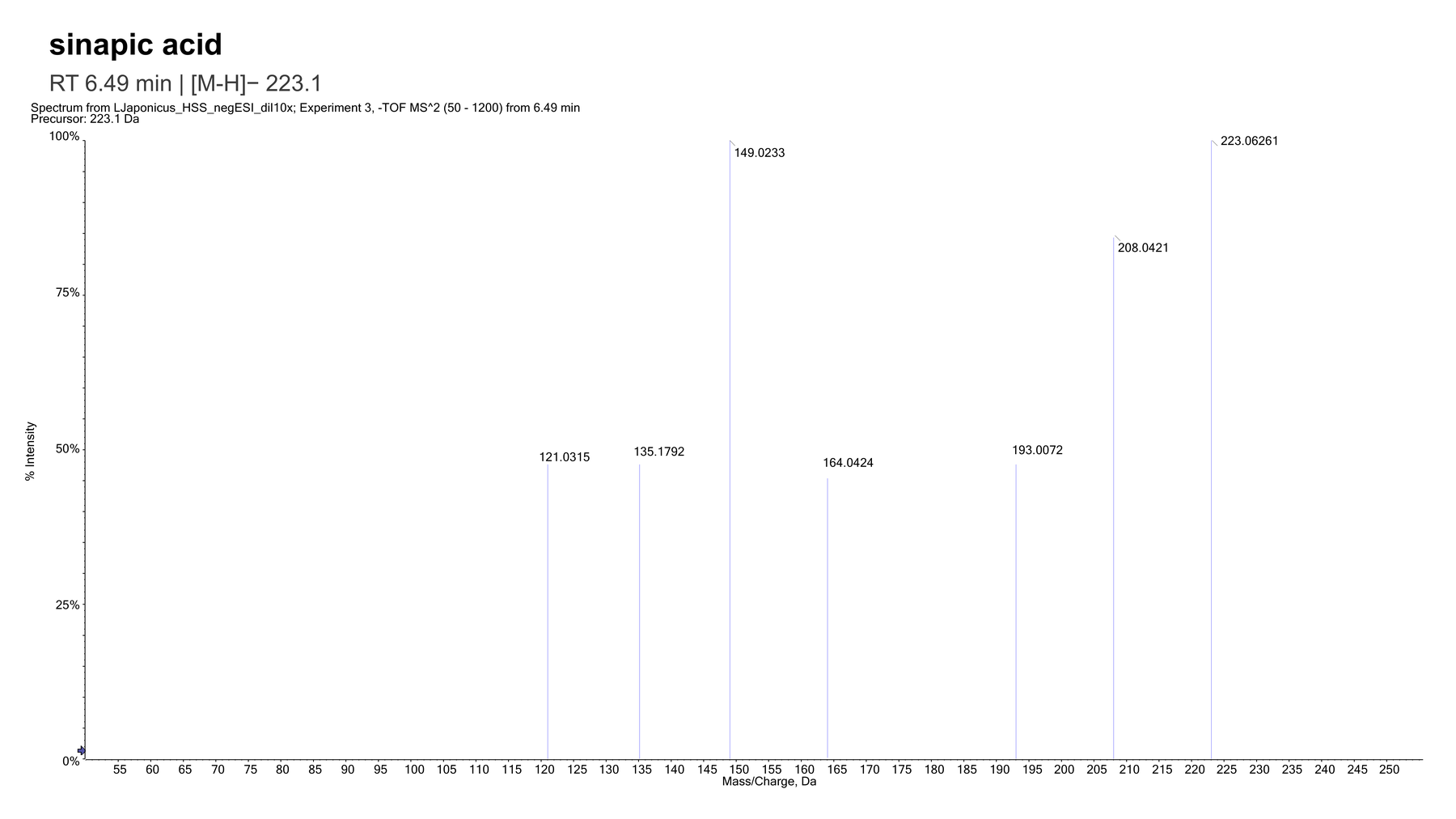


**Figure S9. *Compound 8 – Sinapic acid.*** MS/MS spectrum acquired in ESI− mode at RT 6.49 min for [M−H]⁻ m/z 223.0627. Major annotated fragments: 223.0626, 208.0424, 193.0072, 164.0424, 149.0233, 135.1792, 121.0315.

*[Section A - LC-QTOF-MS/MS]*


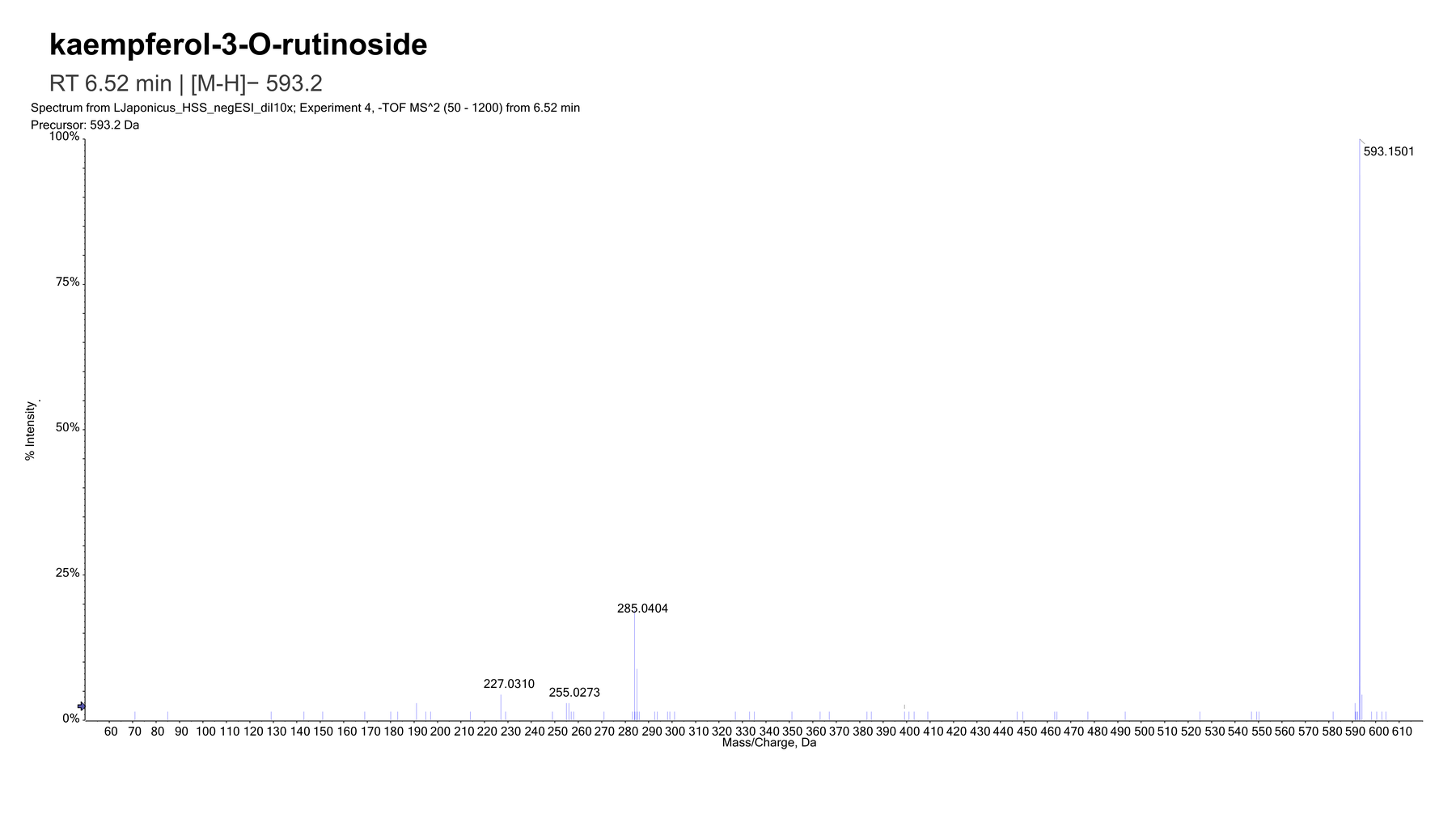


**Figure S10. *Compound 9 – Kaempferol-3-O-rutinoside (Nicotiflorin).*** MS/MS spectrum acquired in ESI− mode at RT 6.52 min for [M−H]⁻ m/z 593.1482. Major annotated fragments: 593.1501, 285.0404, 255.0287, 227.0310.

*[Section A - LC-QTOF-MS/MS]*


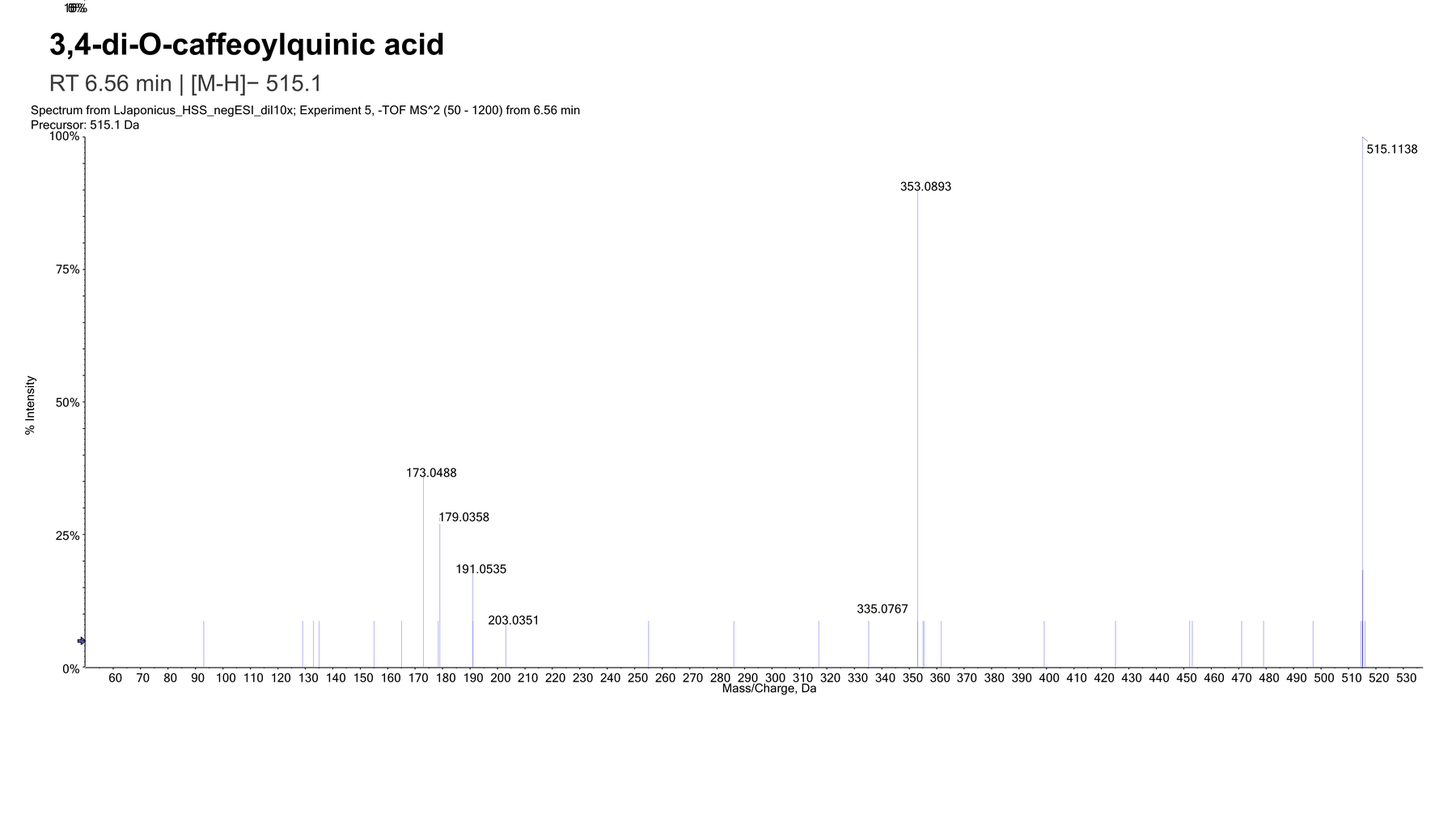


**Figure S11. *Compound 10 – 3,4-Di-O-caffeoylquinic acid.*** MS/MS spectrum acquired in ESI− mode at RT 6.56 min for [M−H]⁻ m/z 515.1214. Major annotated fragments: 515.1138, 353.0893, 335.0767, 203.0351, 191.0535, 179.0358, 173.0488.

*[Section A - LC-QTOF-MS/MS]*


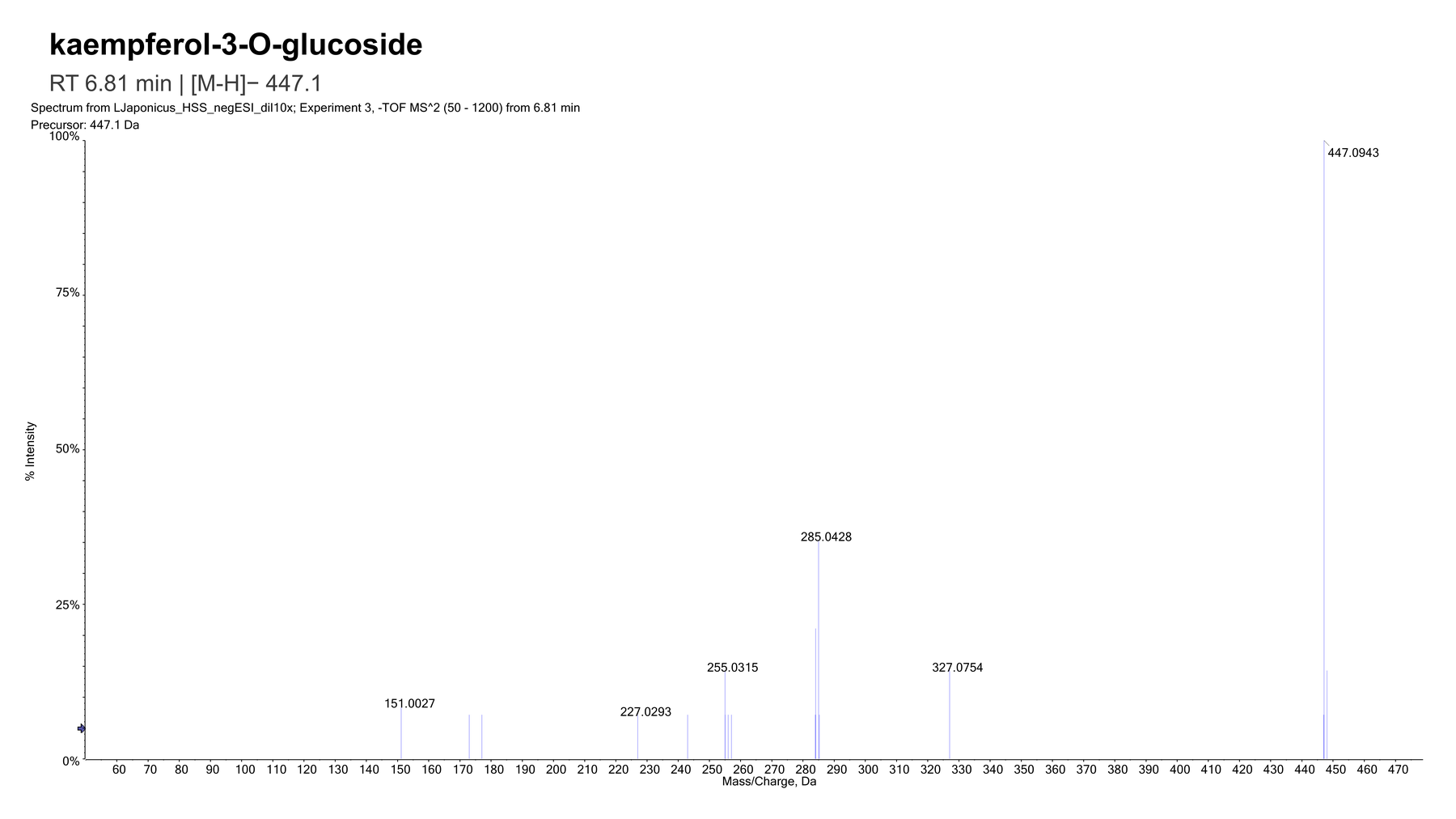


**Figure S12. *Compound 11 – Kaempferol-3-O-glucoside (Astragalin).*** MS/MS spectrum acquired in ESI− mode at RT 6.81 min for [M−H]⁻ m/z 447.0947. Major annotated fragments: 447.0943, 327.0754, 285.0428, 255.0315, 227.0293, 151.0027.

*[Section A - LC-QTOF-MS/MS]*


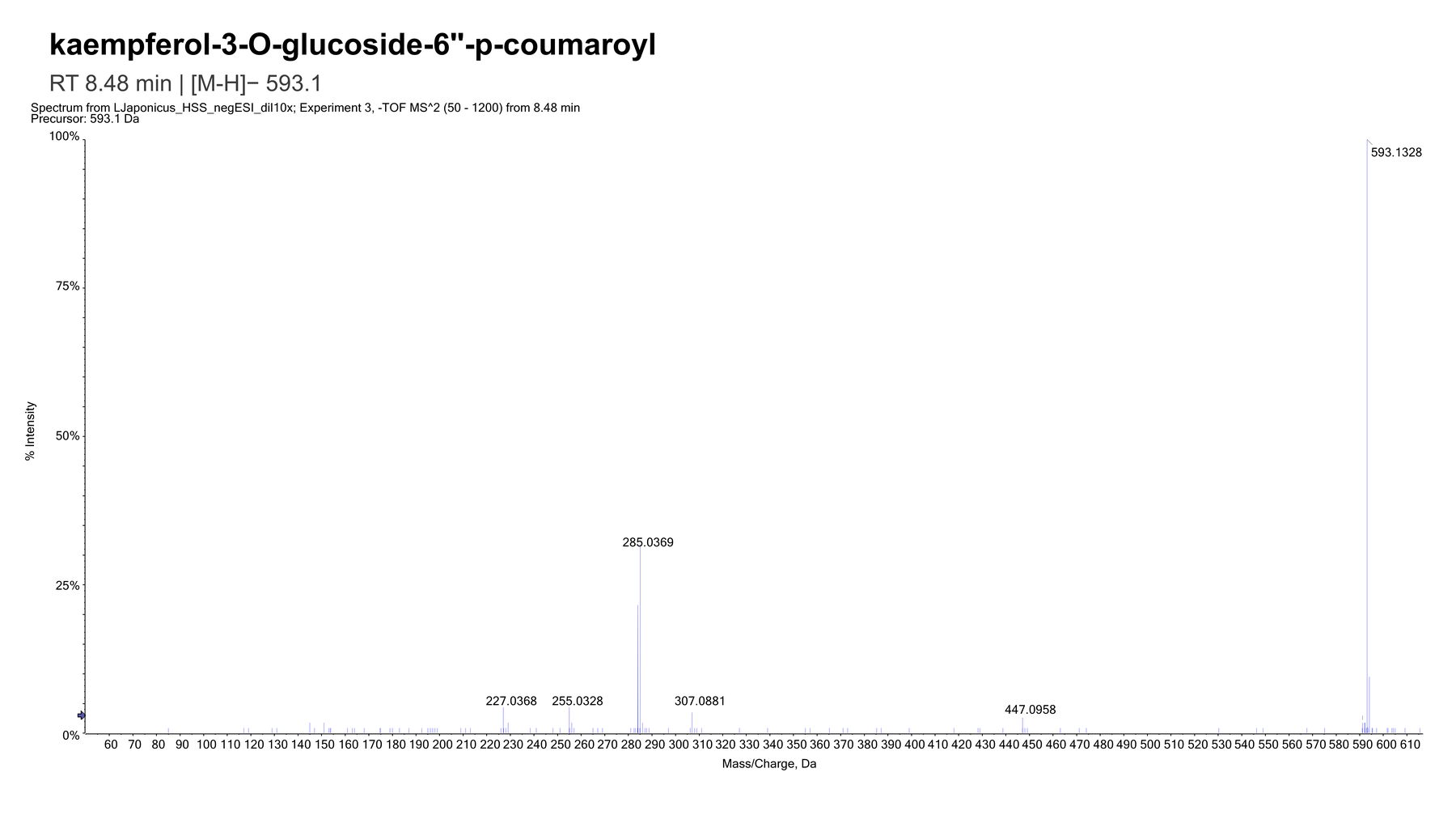


**Figure S13. *Compound 12 – Kaempferol-3-O-glucoside-6′′-p-coumaroyl (Tiliroside).*** MS/MS spectrum acquired in ESI− mode at RT 8.48 min for [M−H]⁻ m/z 593.1318. Major annotated fragments: 593.1328, 447.0958, 307.0881, 285.0369, 255.0328, 227.0368, 145.0283.

*[Section A - LC-QTOF-MS/MS]*


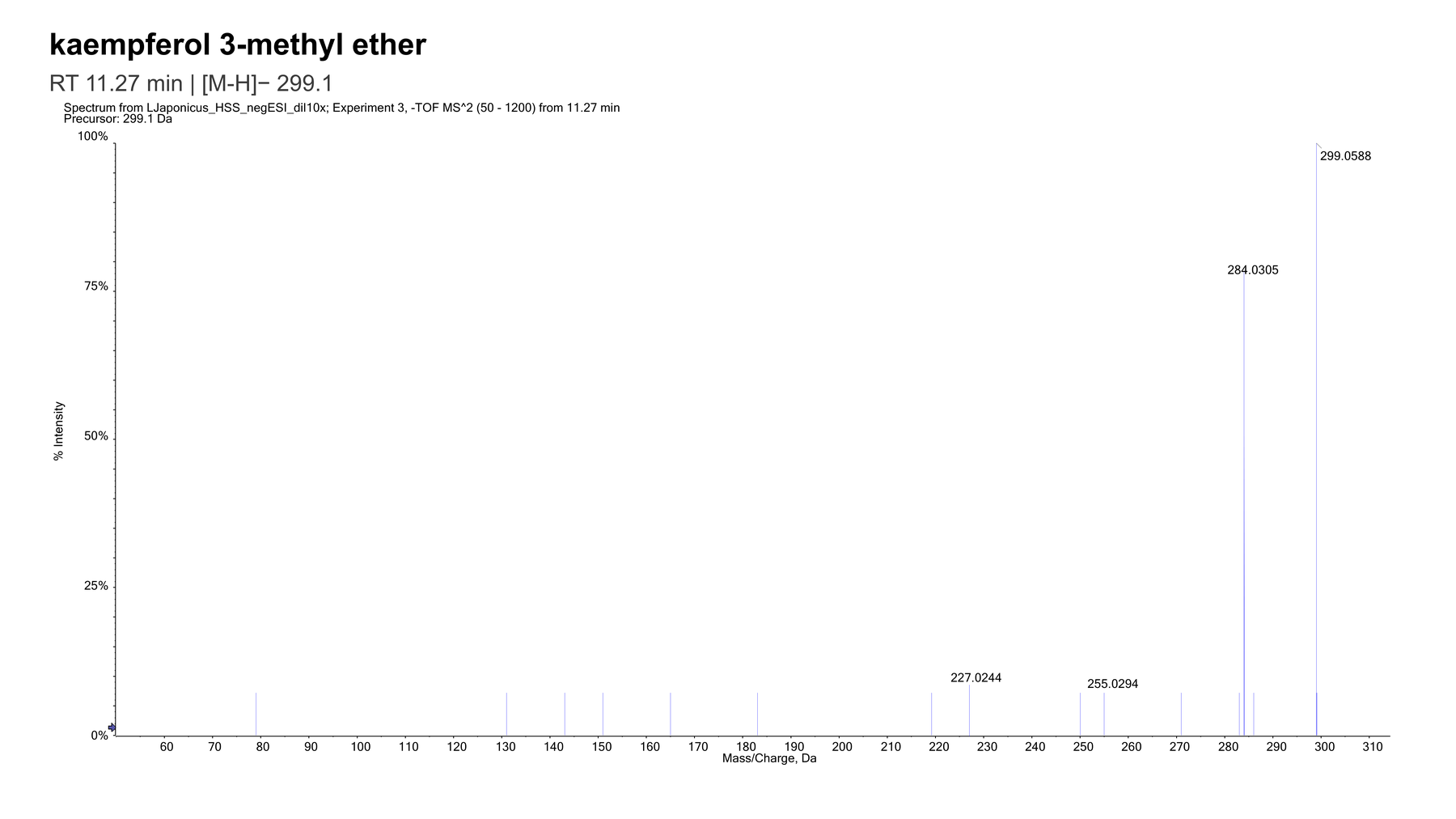


**Figure S14. *Compound 13 – Kaempferol 3-methyl ether.*** MS/MS spectrum acquired in ESI− mode at RT 11.27 min for [M−H]⁻ m/z 299.0588. Major annotated fragments: 299.0556, 284.0305, 255.0294, 227.0244.

━━━━━━━━━━━━━━━━━━━━━━━━━━━━━━━━━━━━━━━━━━

**Section B - NMR SECTION**

*Isolation and structural identification of isoquercitrin*

**Figure S1 (chemical structure), Fig. S1 – Fig. S5 (NMR spectra), Table S1 (NMR data)**

**Note on figure numbering:** *This section uses an independent numbering scheme inherited from the original supplementary material on the isolation and NMR characterization of isoquercitrin. References to "Figure S1", "Fig. S1", "Fig. S2", etc. within Part B refer exclusively to the NMR figures listed below and are distinct from the homonymous figures of Part A (LC-QTOF-MS/MS section).*

━━━━━━━━━━━━━━━━━━━━━━━━━━━━━━━━━━━━━━━━━━

*[Section B - NMR]*

*Isolation and identification of the isoquercitrin the hydro-alcoholic extract of Leonurus japonicus* *aerial part.*

A 200 g portion of the dry hydro-alcoholic extract was solubilized in a methanol (100 mL) : water (1 L) solution and partitioned consecutively against hexane (3 x 200 mL), chloroform (3 x 200 mL), ethyl acetate (3 x 200 mL) and *n*-butanol (3 x 300 mL).

The *n*-butanol fraction (523 mg) was submitted to LH20 column chromatography with hydro-alcoholic (7:3) as eluent to yield fractions F1 (93 mg), F2 (34 mg), F3 (84 mg), F4 (75 mg) and F5 (130 mg). The entire F3 fraction was solubilized in methanol : water (2:8) and submitted to preparative HPLC on a Shimadzu (Kyoto, Japan) system comprising an LC-20AP pump, a manual injector, a DGU-20A5 online degassing unit and a SPD-20A UV-VIS detector with CBM-20A communication bus module, and equipped with a Shimadzu ODS(L) LC-18 preparative column (50 × 25 cm; 5 µm; 100 Å). The mobile phases comprised water [eluent A] and methanol [eluent B] supplied at a flow rate of 20 mL min^−1^ with the following elution profile: a linear gradient from 5% to 100% methanol was applied over 180 minutes. Detection was performed at 350 nm.

The samples collected from preparative HPLC were submitted to analytical HPLC in order to identify, quantify, and purify the individual constituents. Analyses were performed using a Shimadzu system comprising an LC-20 AT pump, a SIL-20A autosampler, a DGU- 20A3 degassing unit, and an SPD-M 20A diode array detector with a CBM-20A module, and equipped with a Phenomenex (Torrance, CA, USA) Luna C18 (250 × 4.6 mm; 5 µm; 100 Å) column. The mobile phases comprised water [eluent A] and methanol [eluent B] supplied at a flow rate of 1.0 mL/min with the following elution profile: gradient from 10 to 66% B (0–32 min), gradient from 66 to 10%(B) (32.01–35 min), and isocratic at 10% B (35.01–45 min). The injection volume was 20 µL, and detection was set at 350 nm. Sample RUB 53 (67 mg) was found to contain a single pure component, while the semi-pure samples Rub 5-76 was further fractionated by preparative HPLC using the chromatographic system described above. The mobile phases comprised water : formic acid (99.7:0.3 v/v) [eluent A] and methanol : acetonitrile (1:1 v/v) [eluent B] supplied at a flow rate of 1.0 mL/min with the following elution profile: gradient from 10 to 100% B (0–90 min) and isocratic at 100% B (90.01–95 min). The injection volume of each sample solution (30 mg/mL) was 500 µL, and detection was set at 350 nm. The chromatographic procedure produced one pure sample labeled RUB 5 (20 mg). The RUB 5 isolated component was dissolved in methanol and submitted to ^1^H, ^13^C, HSQC, HMBC, and 1H-1H COSY NMR for identification. NMR spectra were acquired on a Bruker Daltonics (Billerica, MA, USA) DRX 400 spectrometer operating at 400 MHz for ^1^H NMR and 100 MHz for ^13^C NMR with tetramethylsilane as internal standard.

*Strucutural identification of quercetin 3-O-β-D-glucopyranoside (isoquercitrin).*

The ^1^ H-NMR (S1) and ^13^C-NMR (S2) spectra data of compound isoquercitrin indicated the presence of aglycone quercetin and a glucopyranosil group. The ^1^H-NMR hydrogen signals at δ_H_ 6.29 (d, *J*=2.0 Hz, H8), δ_H_ 6.10 (d, *J*=2.0 Hz, H6), δ_H_ 7.61 (d, *J*=2.0 Hz, H2`), δ_H_ 6.77 (d, *J*=8.5 Hz, H5`) and δ_H_ 7.48 (dd; *J*=8.5; 2.0, H6`) showed, respectively, correlations in the HSQC (S3) spectrum (^1^*J*_CH_) with aromatic carbons at δ_C_ 93.9 (C-8), δ_C_ 99.0 (C6), δ_C_ 116.6 (C2`), δ_C_ 115.1 (C5`) and δ_C_ 122.2 (C6`) of aglycone. It was also observed correlation between anomeric proton signal at δ_H_ 5.15 (d; *J*=7.5 Hz, H1``) with ^13^C signal at δ_C_ 103.4 (C1``) (Table 1). HMBC (S4) and ¹H-¹H-COSY (S5) experiments confirmed the assignment of rings A and B of quercetin. The HMBC correlation was further used to identify the linkage between the aglycone and sugar moiety. The correlation ^3^*J*_CH_ between H-1′′ (*δ*_H_ 5.15) and C-3 (*δ*_C_ 134.7) is a confirmation for the linkage of the sugar moiety to C-3 of the aglycone.  According to the obtained data and its comparison with literature reports (Pokhilo et al.,2021), it was possible to identify quercetin 3-*O*-β-D-glucopyranoside (isoquercitrin).

**Figure S1** quercetin 3-*O*-β-D-glucopyranoside (isoquercitrin)

*[Section B - NMR]*

**Table S1** ^1^H NMR (CD_3_OD; 400 MHz) and ^13^C NMR (CD_3_OD; 100 MHz) data for RUB 5 isolated from the hydro-alcoholic extract of *Leonurus japonicus* aerial part: Isoquercitrin

| Position | Rub 5 (CD_3_OD) | | Isoquercitrin (DMSO)  (Pokhilo, et al., 2021) | |
| --- | --- | --- | --- | --- |
| C | δ_C_ | δ_H_ | δ_C_ | δ_H_ |
| 2 | 157.5 | - | 156.3 | - |
| 3 | 134.7 | - | 133.4 | - |
| 4 | 178.5 | - | 177.5 | - |
| 5 | 162.0 | - | 161.3 | - |
| 6 | 99.0 | 6.10; d; *J*=2.0 | 98.7 | 6.20; d; *J*=2.0 |
| 7 | 164.9 | - | 164.2 | - |
| 8 | 93.9 | 6.29; d; *J*=2.0 | 93.5 | 6.40; d; *J*=2.0 |
| 9 | 158.1 | - | 156.4 |  |
| 10 | 104.7 | - | 104.0 |  |
| 1` | 122.1 | - | 121.2 |  |
| 2` | 116.6 | 7.61; d; *J*=2.0 | 116.3 | 7.58; *J*=2.3 |
| 3` | 148.8 | - | 148.5 |  |
| 4` | 144.9 | - | 144.9 |  |
| 5` | 115.1 | 6.77; d; *J*=8.5 | 115.3 | 6.84; d; *J*=9.0 |
| 6` | 122.2 | 7.48; dd; *J*=8.5; 2.0 | 121.6 | 7.57; dd; *J*=9.0; 2.3 |
| *Glucose moiety* |  |  |  |  |
| 1`` | 103.4 | 5.15 d; *J*=7.5 | 100.9 | 5.46; d; *J*=7.5 |
| 2`` | 74.7 | 3.37-3.40; m | 73.2 | 3,23; m |
| 3`` | 77.1 | 3.32-3.35; m | 76.6 | 3.23; m |
| 4`` | 70.3 | 3.24-3.27; m | 70.0 | 3.10; m |
| 5`` | 77.4 | 3.12-3.15; m | 77.6 | 3.39; m |
| 6`` | 61.6 | 3.60-3.63; m  3.46-3.50; m | 61.0 | 3.57; d; *J*=11.0 |

(^1^H= 500MHz; ^13^C = 125MHz)

d= dublete; dd= duplo dublete; m=multiplete; s= singlete.

Pokhilo, N. D., Fedoreyev, S. A., Tarbeeva, D. V., Veselova, M. V., Grigorchuk, V. P., & Gorovoy, P. G.. Flavonoid Glycosides from the Aerial Part of Lespedeza tomentosa. *Chemistry of Natural Compounds*, 2021, *57*, 1023-1028.

*[Section B - NMR]*

**Fig. S1** ^1^H NMR (CD_3_OD; 400 MHz) spectrum of compound RUB 5 isolated from the hydro-alcoholic extract of *Leonurus japonicus* aerial part: Isoquercitrin

*[Section B - NMR]*

**Fig. S2** ^13^C NMR (CD_3_OD; 100 MHz) spectrum of compound RUB 5 isolated from the hydro-alcoholic extract of *Leonurus japonicus* aerial part: Isoquercitrin

*[Section B - NMR]*

**Fig. S3** HSQC spectrum of compound RUB 5 isolated from the hydro-alcoholic extract of *Leonurus japonicus* aerial part: Isoquercitrin

*[Section B - NMR]*

**Fig. S4** HMBC spectrum of compound RUB 5 isolated from the hydro-alcoholic extract of *Leonurus japonicus* aerial part: Isoquercitrin

*[Section B - NMR]*

**Fig. S5** COSY spectrum of compound RUB 5 isolated from the hydro-alcoholic extract of *Leonurus japonicus* aerial part: Isoquercitrin
